# Development of dual aptamers-functionalized c-MET PROTAC degraders for targeted therapy of osteosarcoma

**DOI:** 10.7150/thno.99588

**Published:** 2025-01-01

**Authors:** Xuekun Fu, Jie Huang, Xinxin Chen, Duoli Xie, Hongzhen Chen, Zhijian Liang, Zhuqian Wang, Yiying Liang, Aiping Lu, Chao Liang

**Affiliations:** 1 Department of Systems Biology, School of Life Sciences, Southern University of Science and Technology, Shenzhen 518055, China.; 2 Institute of Integrated Bioinfomedicine and Translational Science (IBTS), School of Chinese Medicine, Hong Kong Baptist University, Hong Kong SAR 999077, China.; 3 Shenzhen LingGene Biotech Co., Ltd., Shenzhen 518055, China.; 4 Guangdong-Hong Kong-Macau Joint Lab on Chinese Medicine and Immune Disease Research, Guangzhou 510006, China.; 5 Shanghai University of Traditional Chinese Medicine, Shanghai 200032, China.; 6 State Key Laboratory of Proteomics, National Center for Protein Sciences (Beijing), Beijing Institute of Lifeomics, Beijing 100850, China.

**Keywords:** nucleolin, c-MET, PROTAC, AS1411, osteosarcoma

## Abstract

**Rationale:** Osteosarcoma (OS) is the most common bone malignancy. c-MET is recognized as a therapeutic target. However, traditional c-MET inhibitors show compromised efficacy due to the acquired resistance and side effects. PROTACs targeting c-MET have displayed improved antitumor efficacy by overcoming drug resistance, whereas safety concern caused by lack of tumor-targeting ability is still a pending issue. AS1411 is an aptamer that recognizes and penetrates tumors by targeting nucleolin (NCL) overexpressed on the surface of tumor cells. Since NCL interacts with an E3 ligase MDM2 intracellularly, we repurposed AS1411 as an MDM2 recruiter by employing NCL as a bridge.

**Methods:** We select the ssDNA c-MET aptamer SL1 as the c-MET ligand to design dual aptamer-functionalized PROTACs, as SL1 can be easily conjugated to AS1411 through base-pair complementarity using a nucleic acid linker. Four AS1411-SL1 chimeras are generated by linking AS1411 to either the 5' or 3' terminus of SL1 via two different lengths of nucleic acid linkers. The therapeutic efficacy of these PROTACs is evaluated through both* in vitro* and* in vivo* experiments.

**Results:** The PROTACs enable the ubiquitination and degradation of c-MET. The PROTACs effectively inhibit growth, enhance apoptosis, and overcome drug resistance of OS cells *in vitro*. The PROTACs demonstrate *in vivo* tumor-targeting ability and facilitate the OS treatment with no detectable toxicity.

**Conclusion:** This study suggests that the AS1411-SL1 chimeras could be promising c-MET degraders for targeted therapy of OS.

## Introduction

Osteosarcoma (OS), the most prevalent primary bone tumor in children and adolescents, originates from bone-forming mesenchymal stem cells 1. It typically manifests in the metaphysis of long bones, with less common occurrences in the spine, pelvis, and sacrum 2. The malignancy of OS is characterized by its highly aggressive nature, rapid progression, and the severe pain associated with bone destruction 3-5. Before 1970, OS treatment primarily relied on surgical resection, with 5-year survival rates below 20%. Currently, the standard treatment involves a combination of surgery and chemotherapy. Rarely, radiation therapy is also an option if OS patients exhibit poor response to the standard treatment. Despite the intensive combination of surgery and chemotherapy, limited therapeutic progress has been made and the five-year survival rate of OS patients remains suboptimal, with nearly one-third of patients suffering from recurrence or progression 6. This is mainly attributed to the tendency of OS to metastasize and its acquired resistance to conventional chemotherapeutic drugs 7, 8. Recent studies have shed light on novel therapeutic targets for OS, including vascular endothelial growth factor (VEGF), insulin-like growth factor-1 receptor (IGF1R), human epidermal growth factor receptor 2 (HER2), mammalian target of rapamycin (mTOR), receptor activator of nuclear factor kappa-B ligand (RANKL), and platelet-derived growth factor (PDGF) 9. Nonetheless, clinical research has lagged, and drugs against these targets have yet to demonstrate substantial benefits 10. Therefore, there is an urgent need to develop new therapeutic strategies to enhance the treatment outcomes for OS patients 11.

C-mesenchymal-epithelial transition factor (c-MET) is a transmembrane receptor tyrosine kinase that belongs to the MET family 12. Hepatocyte growth factor (HGF) is the natural ligand for c-MET 13. Upon binding with HGF, c-MET triggers a series of intracellular downstream signaling cascades, such as extracellular signal-regulated kinase (ERK), and phosphoinositide 3-kinase-AKT axis (PI3K/AKT), leading to pleiotropic biological responses such as cell proliferation, scattering and motility, invasion, angiogenesis, and morphological changes 14. Overexpression or persistent activation of c-MET has been observed in various malignancies 12. Small molecular inhibitors of c-MET, such as foretinib, crizotinib, tepotinib, cabozantinib, and capmatinib, have been approved or under clinical trials for treating non-small cell lung cancer, gastric cancer, pancreatic adenocarcinoma, and hepatocellular carcinoma 15, 16. Recently, dysfunction of c-MET has also been highly associated with OS initiation, progression, invasion, and metastasis 17. Intriguingly, c-MET was initially discovered as the protein product of the translocated promoter region (TPR)-MET transforming oncogene derived from OS cells 18. c-MET inhibition strategies have been experimentally tested and showed encouraging results in inhibiting malignant properties of OS cells *in vitro* and xenograft growth *in vivo* 17. Nonetheless, two critical hurdles need to be addressed before contemplating the clinical use of c-MET inhibitors for OS treatment. Firstly, the efficacy of the c-MET inhibitors is frequently compromised by acquired resistance stemming from c-MET mutations 19. Secondly, the c-MET inhibitors are incapable of distinguishing tumor cells from normal cells, which leads to serious adverse effects since c-MET is fundamentally expressed in normal tissues, where it plays a role in a range of physiological functions including embryonic development, tissue regeneration, and wound healing 20-24.

Proteolysis targeting chimeras (PROTACs) have been increasingly recognized for their potential to modulate proteins of interest (POIs) 25. Structurally, they are heterobifunctional molecules composed of two moieties connected by a linker. One moiety selectively binds to a POI, while the second moiety recruits an E3 ubiquitin ligase 26. The simultaneous engagement of the POI and the E3 ligase by the PROTAC molecule initiates the ubiquitination of the POI, resulting in the subsequent degradation of the POI by the ubiquitin-proteasome system. PROTACs provide a powerful approach to overcoming drug resistance 27. Arvinas, for instance, has developed an estrogen receptor (ER)-targeting PROTAC named ARV-471. This molecule, which links an ER-binding ligand to a cereblon (CRBN) E3 ligase recruiter, is capable of degrading both wild-type ERα and ERα mutants (Y537S and D538G) 28. To date, single and polyclonal *MET* kinase domain mutations in codons H1094, G1163, L1195, D1228, and Y1230 and high levels of amplification of the *MET* exon 14-mutant allele have been found to drive resistance to c-MET inhibitors 29. PROTACs targeting c-MET have been developed to degrade mutant c-MET via conjugating c-MET inhibitors with recruiters for E3 ligases including CRBN and Von Hippel-Lindau (VHL) 30-32. Impressively, these PROTACs show improved antitumor efficacy by overcoming the acquired resistance associated with c-MET mutation and display better tolerance than conventional c-MET inhibitors 30-32. Despite these advancements, the issue of non-selectivity remains, as these PROTACs are not tumor-specific and do not fully alleviate the safety concerns associated with conventional c-MET inhibitors. Therefore, the development of tumor-targeting c-MET PROTAC degraders that harmonize therapeutic effectiveness with an improved safety profile continues to be a critical goal 33.

Aptamers are single-stranded DNAs (ssDNAs) or RNAs that exhibit a remarkable capacity to bind to various targets, including proteins, peptides, carbohydrates, small molecules, toxins, and even intact cells 34. Aptamers are structurally adaptable, often forming helices and loops, which enable them to adopt diverse shapes necessary for target interaction. The selection of aptamers is achieved through the systematic evolution of ligands by exponential enrichment (SELEX), a process that screens a vast oligonucleotide library. This iterative technique progressively eliminates non-binding sequences while multiplying those that bind to the target. Enhancing the specificity of aptamer candidates may involve initial positive selection stages followed by negative selection rounds. To ensure the oligonucleotide pool is rich in high-affinity aptamers, multiple cycles of SELEX are conducted, each with greater selectivity pressure 35. AS1411 is a 26-base quadruplex-forming ssDNA aptamer that has been widely used as a tumor-specific and -penetrating ligand. The target of AS1411 is nucleolin (NCL), which acts as a shuttling protein to carry ribosomal protein from the cytoplasm to the nucleus during the assembly of ribosomes in physiological conditions 36. However, in tumor cells, NCL is frequently overexpressed and specifically translocated onto the cell surface, functioning as a receptor for AS1411-mediated tumor-specific delivery and internalization of therapeutics 37, 38. Moreover, NCL within tumor cells has been shown to be a binding partner of mouse double minute 2 homolog (MDM2), which is a commonly used E3 ligase harnessed by PROTACs 39. Inspired by these findings, we recently demonstrated that AS1411, after selectively entering tumor cells, can recruit MDM2 via forming an NCL-bridged complex, *i.e.*, AS1411-NCL-MDM2 40. Thus, we hypothesize that AS1411, when conjugated to a c-MET ligand, facilitates the design of tumor-targeting PROTAC degraders for c-MET.

In this study, we choose an ssDNA aptamer SL1 as the c-MET ligand 41 to create the dual aptamers-functionalized PROTACs since SL1 could be readily conjugated to AS1411 using a nucleic acid linker through base-pair complementarity, which is a simpler approach than the covalent coupling of AS1411 with traditional c-MET inhibitors. We generate four AS1411-SL1 chimeras by connecting AS1411 to either the 5' or 3' terminus of SL1 via two lengths of the nucleic acid linkers. Neither AS1411 nor SL1 alone influences the expression of c-MET protein, whereas the AS1411-SL1 chimeras orchestrate the formation of an MDM2-NCL-PROTAC-c-MET quaternary complex that triggers the ubiquitination and degradation of c-MET. *In vitro* experiments reveal that the chimeras selectively fight against OS cells and overcome drug resistance. After systemic administration, the chimeras show OS-specific distribution and potent antitumor activity without observable toxicity *in vivo*. In essence, the AS1411 and SL1-functionalized c-MET PROTACs offer a promising targeted therapeutic strategy for OS that optimizes efficacy while minimizing safety concerns.

## Results

### Design of c-MET PROTAC degraders consisting of AS1411 and SL1

**Figure 1:**
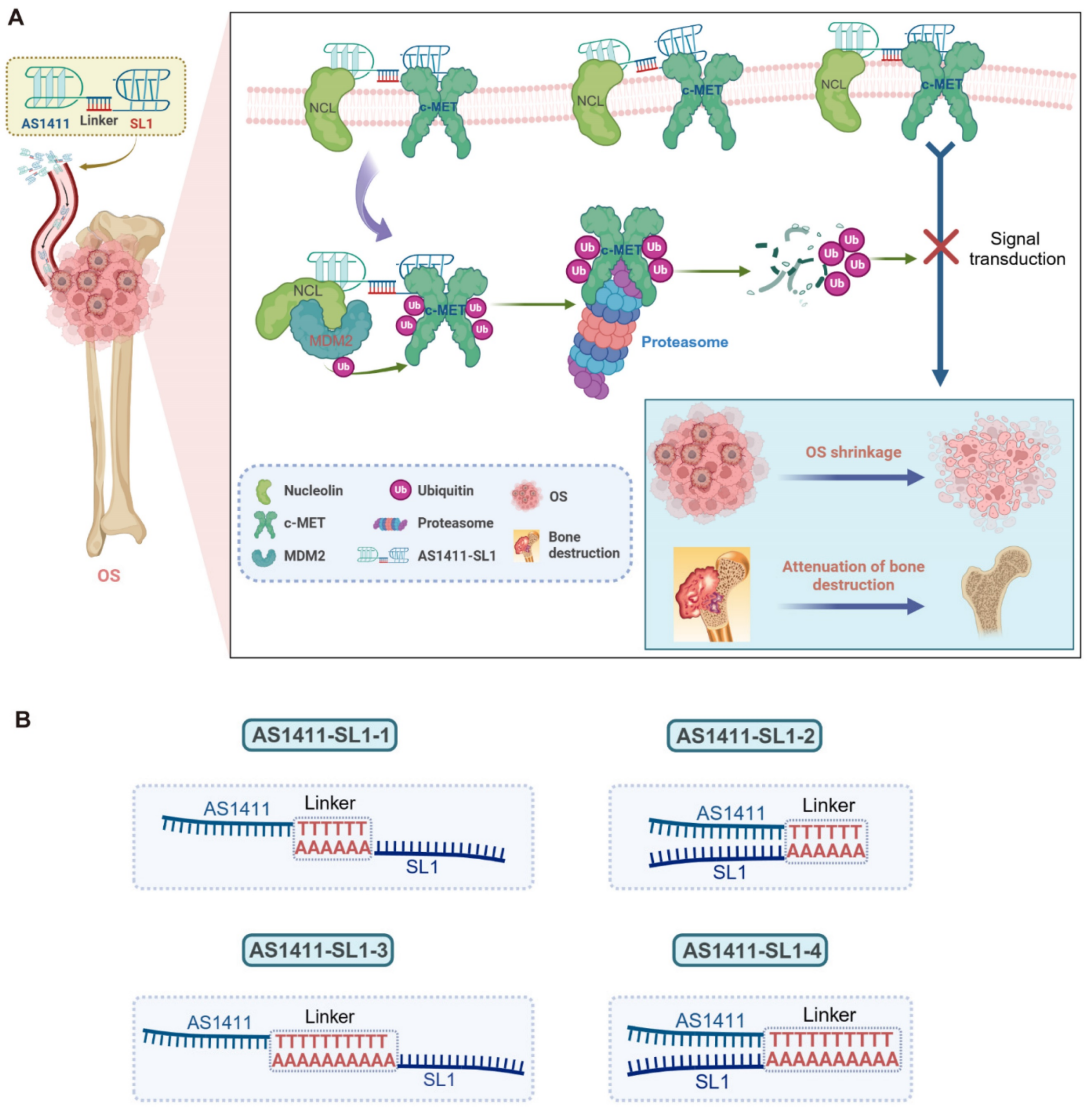
** Design of the dual aptamers-functionalized c-MET PROTACs.** (**A**) A schematic illustration showing the design of the dual aptamers-functionalized c-MET PROTACs and the proposed action of mode of the PROTACs. Briefly, AS1411 would be conjugated with SL1 via a double-stranded DNA linker, resulting in the formation of the AS1411-SL1 chimeras. The AS1411-SL1 chimeras would selectively recognize OS cells via targeting cell surface NCL and be internalized into OS cells. Upon entering OS cells, the AS1411-SL1 chimeras would bring the NCL-MDM2 complex into close proximity with c-MET, enabling the transfer of ubiquitin molecules to c-MET and proteasomal degradation of c-MET. The degradation of c-MET induced by the AS1411-SL1 chimeras could effectively inhibit the downstream signal activation of c-MET, resulting in OS shrinkage and attenuation of OS-induced bone destruction. (**B**) A schematic diagram showing the conjugation of AS1411 with SL1 through a double-stranded DNA linker. AS1411 would be linked with either 5' or 3' terminus of SL1 via a DNA linker consisting of either 6 or 10 adenine-thymine (A-T) base pairs to generate four PROTAC molecules: AS1411-SL1-1, AS1411-SL1-2, AS1411-SL1-3, and AS1411-SL1-4.

To develop c-MET PROTAC degraders, AS1411 would be linked with the c-MET-specific aptamer SL1 through a double-stranded DNA linker. The engineered AS1411-SL1 chimeras would selectively bind to surface NCL overexpressed on OS cells and be internalized into the OS cells. Once inside OS cells, the AS1411-SL1 chimeras would recruit the NCL-MDM2 complex into close proximity with the c-MET protein, triggering ubiquitination and subsequent degradation of c-MET. This targeted degradation of c-MET by the AS1411-SL1 chimeras would effectively disrupt downstream signaling pathways, resulting in OS shrinkage and attenuation of OS-induced bone destruction (**Figure 1A**). The construction of the AS1411-SL1 chimeras would involve connecting AS1411 to either the 5' or 3' terminus of SL1 through a DNA linker composed of 6 or 10 adenine-thymine (A-T) base pairs. This would yield four PROTAC chimeras: AS1411-SL1-1, AS1411-SL1-2, AS1411-SL1-3, and AS1411-SL1-4 (**Figure 1B**).

### Characterization and tumor-targeting ability of AS1411-SL1 chimeras *in vitro*

We characterized the AS1411-SL1 chimeras by non-denaturing polyacrylamide gel electrophoresis (PAGE). Our results showed the stable conjugation of AS1411 with SL1 in the chimeras (**Figure S1A**). We incubated an IgG isotype or anti-NCL antibody with an OS cell line MNNG/HOS, an osteoblast precursor cell line MC3T3-E1, an osteocyte-like cell line MLO-Y4, and a non-malignant epithelial cell line MCF 10A. Flow cytometric analysis demonstrated that there was an abundant expression of NCL on the surface of MNNG/HOS cells rather than on MC3T3-E1, MLO-Y4, or MCF 10A cells (**Figure S1B**). We tested the binding affinity of a cytosine-rich oligonucleotide (CRO, negative control), AS1411, SL1, AS1411-SL1-1, AS1411-SL1-2, AS1411-SL1-3, and AS1411-SL1-4 with MNNG/HOS cells by flow cytometry. AS1411, SL1, and all four AS1411-SL1 chimeras showed elevated binding ability with MNNG/HOS cells than CRO, which might be attributed to the high levels of both NCL and c-MET present on the surface of OS cells. Notably, AS1411 exhibited better binding ability than SL1, suggesting a greater abundance of NCL than c-MET on OS cells. The four chimeras displayed higher binding ability than either AS1411 or SL1, likely due to the synergistic action of AS1411 and SL1. Among the four chimeras, AS1411-SL1-2 and AS1411-SL1-3 showed the most promising targeting capabilities for MNNG/HOS cells (**Figure S1C**). Confocal imaging showed that all four AS1411-SL1 chimeras and AS1411 were internalized into MNNG/HOS cells, whereas SL1 merely anchored to the cell membrane and did not enter the OS cells (**Figure S1D**), suggesting that AS1411 facilitated the cellular uptake of the chimeras. Serum stability assay showed that all four AS1411-SL1 chimeras were slightly degraded but still visible for 24 h in 10% serum *in vitro* (**Figure S1E**). We incubated AS1411-SL1 chimeras with the MNNG/HOS cell lysates and showed that AS1411-SL1 chimeras were stable, suggesting that 6 A-T base pairs were sufficient to maintain the conjugation of AS1411 with SL1 (**Figure S1E**).

### Degradation of c-MET induced by the AS1411-SL1 chimeras *in vitro*

**Figure 2:**
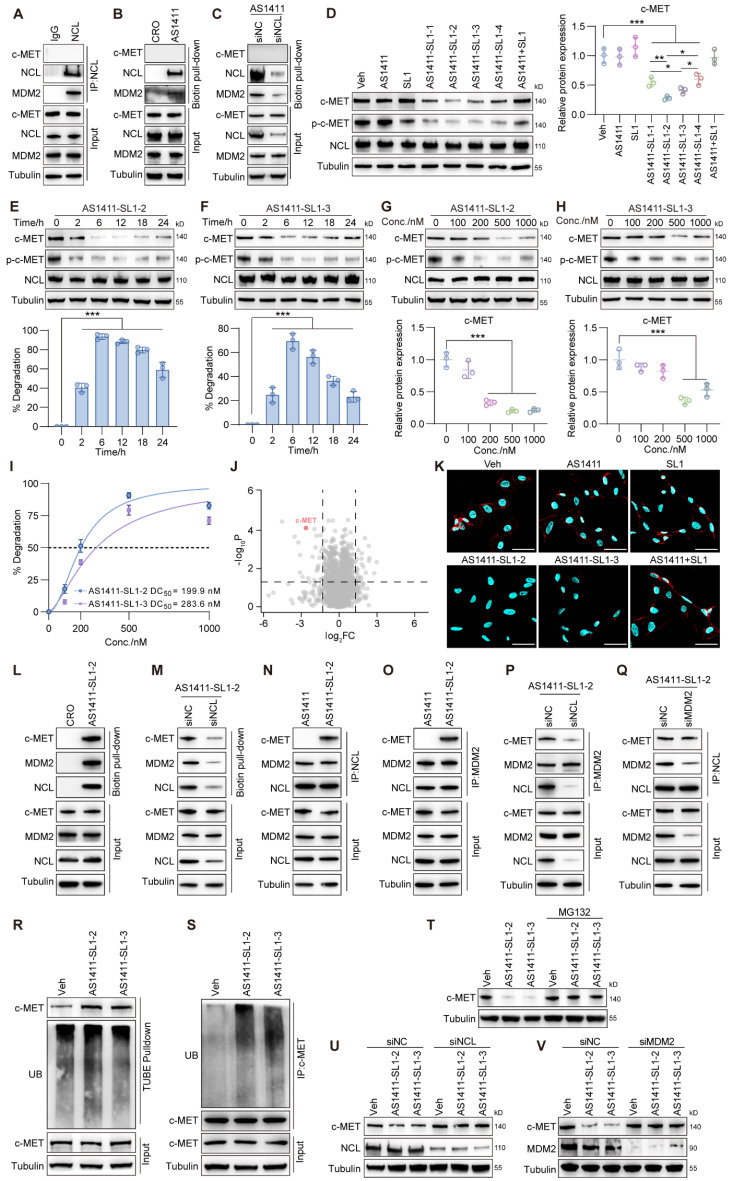
** The AS1411-SL1 chimeras-mediated ubiquitination and degradation of c-MET.** (**A**) Co-immunoprecipitation (IP) of whole-cell extracts from a human *OS cell line* MNNG/HOS using an IgG or an anti-NCL antibody. (**B**) Pull-down assay of whole-cell extracts from MNNG/HOS cells after treatment with 500 nM biotin-labeled cytosine-rich oligonucleotides (CRO, a negative control) or AS1411 for 6 h using streptavidin-coated magnetic beads. (**C**) Pull-down assay of whole-cell extracts from siNC- or siNCL-transfected MNNG/HOS cells after treatment with 500 nM biotin-labeled AS1411 for 6 h. (**D**) Levels of c-MET, p-c-MET, and NCL in MNNG/HOS cells after treatment with vehicle (Veh, PBS), AS1411, SL1, AS1411-SL1-1, AS1411-SL1-2, AS1411-SL1-3, AS1411-SL1-4 or AS1411+SL1 (a physical combination of AS1411 and SL1) at a concentration of 500 nM for 6 h. Left, western blotting images; right, quantification of the c-MET protein. (**E** and** F**) Levels of c-MET, p-c-MET, and NCL in MNNG/HOS cells treated with 500 nM AS1411-SL1-2 (**E**) or AS1411-SL1-3 (**F**) at the indicated time points (0, 2, 6, 12, 18, and 24 h). Top, western blotting images; bottom, quantification of the c-MET degradation. (**G** and **H**) Levels of c-MET, p-c-MET, and NCL in MNNG/HOS cells treated with AS1411-SL1-2 (**G**) or AS1411-SL1-3 (**H**) at the indicated concentrations (0, 100, 200, 500, and 1000 nM). Top, western blotting images; bottom, quantification of the c-MET protein. (**I**) The DC_50_ values for AS1411-SL1-2 and AS1411-SL1-3. The data were analyzed by fitting a curve using nonlinear regression. (**J**) Quantitative proteomics showing selective c-MET degradation after treatment with 500 nM AS1411-SL1-2 for 6 h. Cut-off false discovery rate (FDR) < 0.1 and |log_2_FC| ≥ 1. (**K**) Immunofluorescent staining showing c-MET expression on the surface of MNNG/HOS cells after the indicated treatments for 12 h. Nuclei of MNNG/HOS cells were counterstained with DAPI. Cell surface c-MET protein was shown in red. Cell nuclei were shown in blue. Scale bar = 50 μm. (**L**) Pull-down assay of whole-cell extracts from MNNG/HOS cells after treatment with 500 nM biotin-labeled CRO or AS1411-SL1-2 for 6 h. (**M**) Pull-down assay of whole-cell extracts from siNC- or siNCL-transfected MNNG/HOS cells after treatment with 500 nM biotin-labeled AS1411-SL1-2 for 6 h. (**N** and** O**) Co-IP of whole-cell extracts from MNNG/HOS cells treated with 500 nM AS1411 or AS1411-SL1-2 for 2 h using an anti-NCL antibody (**N**) or an anti-MDM2 antibody (**O**). (**P** and** Q**) Co-IP of whole-cell extracts from siNCL (**P**)- or siMDM2 (**Q**)-transfected MNNG/HOS cells after treatment with 500 nM AS1411-SL1-1 for 2 h. (**R**) Ubiquitination of c-MET in MNNG/HOS cells treated with Veh or 500 nM AS1411-SL1-2 or AS1411-SL1-3 for 6 h with the presence of 10 μM MG132, as determined by the tandem ubiquitin-binding entities (TUBEs) conjugated to magnetic beads. (**S**) c-MET ubiquitination in MNNG/HOS cells treated with Veh or 500 nM AS1411-SL1-2 or AS1411-SL1-3 for 6 h with the presence of 10 μM MG132, as determined by Co-IP assay. (**T**) Level of c-MET in MNNG/HOS cells after treatment with Veh or 500 nM AS1411-SL1-2 or AS1411-SL1-3 for 6 h, in the presence or absence of 10 μM proteasome inhibitor MG132. (**U** and **V**) Level of c-MET protein in siNCL- (**U**) or siMDM2- (**V**) transfected MNNG/HOS cells after treatment with Veh or 500 nM AS1411-SL1-2 or AS1411-SL1-3 for 6 h. Data were presented as mean ± SD. Each of the above experiments was repeated three times. *P*-values from one-way ANOVA: *P < 0.05, **P < 0.01, ***P < 0.001.

Before testing whether AS1411-SL1 chimeras could induce the c-MET degradation, we performed co-immunoprecipitation (Co-IP) and showed that NCL interacted with MDM2 rather than c-MET in MNNG/HOS cells (**Figure 2A**). We investigated if NCL was a substrate for MDM2. Silencing MDM2 did not result in increased NCL expression, indicating that NCL is not a substrate for MDM2 (**Figure S2A**). We incubated MNNG/HOS cells with biotin-labeled AS1411 and pull-down assay demonstrated that AS1411 captured NCL and MDM2 but not c-MET (**Figure 2B**). We silenced NCL expression and observed that AS1411 could not effectively capture MDM2 in NCL-deficient MNNG/HOS cells, emphasizing that AS1411 recruited MDM2 in an NCL-dependent manner (**Figure 2C**). We evaluated the effects of the chimeras on c-MET mRNA and protein expression and phosphorylation (p-c-MET). All four chimeras did not affect the mRNA expression of c-MET in MNNG/HOS cells (**Figure S2B**). However, they induced significant c-MET degradation and inhibition of p-c-MET, when compared to AS1411, SL1, and AS1411+SL1 (a physical combination of AS1411 with SL1) (**Figure 2D**). Notably, AS1411-SL1-2 and AS1411-SL1-3 demonstrated superior efficacy in degrading c-MET and inhibiting c-MET phosphorylation compared to other chimeras (**Figure 2D**). A time-dependent decrease in c-MET level and c-MET phosphorylation were observed in MNNG/HOS cells treated with AS1411-SL1-2 or AS1411-SL1-3 (**Figure 2E and 2F**). AS1411- SL1-2 and AS1411- SL1-3 enhanced c-MET degradation and inhibition of c-MET phosphorylation with increasing concentrations (**Figure 2G and 2H**). The level of NCL was not altered in MNNG/HOS cells after treatment with varied concentrations and incubation times of the AS1411-SL1 chimeras (**Figure 2D-2H**). DC_50_ (concentration that results in a 50% protein degradation) for AS1411-SL1-2 and AS1411-SL1-3 in MNNG/HOS cells was 199.9 nM and 283.6 nM, respectively (**Figure 2I**). We also demonstrated the effective degradation of c-MET by the AS1411-SL1 chimeras in a human prostate cancer cell line DU145 (DC_50_ for AS1411-SL1-2 and AS1411-SL1-3 was 237.4 nM and 287.8 nM, respectively), and a human cervical cancer cell line HeLa (DC_50_ for AS1411-SL1-2 and AS1411-SL1-3 was 575.2 nM and 337.1 nM, respectively) (**Figure S2C-S2N**). To demonstrate the selective degradation capability of the AS1411-SL1 chimeras, we assessed the level of MST1R, a homolog of c-MET 42, in MNNG/HOS cells following treatment with the AS1411-SL1 molecules. None of the four AS1411-SL1 chimeras triggered the degradation of MST1R (**Figure S2O**). Our proteomic analysis revealed that AS1411-SL1-2 decreased c-MET expression and also altered the abundance of numerous proteins that function as potential downstream effectors of c-MET or are involved in the broad spectrum of biological responses orchestrated by c-MET signaling (**Figure 2J and Table S1**). We determined whether the AS1411-SL1 chimeras influenced the downstream signaling activation of c-MET. There were decreased levels of phosphorylated AKT and ERK (p-AKT and p-ERK) in MNNG/HOS cells upon treatment with AS1411-SL1-2 (**Figure S2P**). Moreover, immunofluorescent images and flow cytometric analysis revealed that surface expression of c-MET on MNNG/HOS cells was reduced upon treatment with AS1411-SL1-2 or AS1411-SL1-3 when compared to the cells incubated with AS1411, SL1, or AS1411+SL1 (**Figure 2K and Figure S2Q**). We assessed the effectiveness of AS1411-SL1-2 in reducing the c-MET level in the Hs746T cells harboring the D1228N mutation, which exhibited resistance to the c-MET inhibitor Tepotinib^32^. Our findings indicated that AS1411-SL1-2 markedly diminished c-MET expression in these cells in a dose-dependent manner (**Figure S2R**). Furthermore, we demonstrated that AS1411-SL1-2 exhibited comparable efficacy in degrading c-MET to PRO-6E, a previously developed c-MET degrader 43 (**Figure S2R**). In a previous study, AS1411 was conjugated with SL1, pioneering a novel approach known as a bispecific aptamer-induced artificial protein-pairing (BAAP) strategy aimed at impeding c-MET phosphorylation 44. Employing AS1411-SL1-2 at a concentration of 100 nM, we were able to replicate these findings by modulating the level of phosphorylated c-MET without altering the expression of c-MET itself (**Figure S2S**). We also tested the effects of the AS1411-SL1 chimeras on the level of c-MET in non-malignant MCF 10A cells. AS1411-SL1-2 and AS1411-SL1-3 showed no impact on the c-MET level in MCF 10A cells (**Figure S2T**), underscoring the tumor selectivity of the AS1411-SL1 chimeras.

### Assembly of an MDM2-NCL-PROTAC-c-MET quaternary complex

We incubated MNNG/HOS cells with AS1411-SL1-2 and showed that AS1411-SL1-2 captured c-MET along with NCL and MDM2 (**Figure 2L**). We silenced NCL and observed decreased levels of c-MET and MDM2 captured by AS1411-SL1-2 (**Figure 2M**). In the presence of AS1411-SL1-2, both c-MET and MDM2 were trapped with NCL in MNNG/HOS cells (**Figure 2N**). We also showed the efficient trapping of NCL and c-MET with MDM2 in MNNG/HOS cells incubated with AS1411-SL1-2 (**Figure 2O**). Silencing of NCL attenuated the AS1411-SL1-2-mediated trapping of c-MET with MDM2, whereas silencing of MDM2 did not impact the trapping of c-MET with NCL by AS1411-SL1-2 (**Figure 2P and 2Q**). Surface plasmon resonance (SPR) experiments showed that AS1411 had high binding affinity with NCL rather than MDM2 (**Figure S2U**), and AS1411-SL1-2 directly interacted with NCL and c-MET but not MDM2 (**Figure S2V**). Confocal imaging demonstrated that AS1411-SL1-2 induced the co-localization of c-MET with NCL and MDM2 (**Figure S2W**). Pull-down assays in combination with enzyme-linked immunosorbent assay (ELISA) quantitatively showed that AS1411 only captured NCL and MDM2 and SL1 only trapped c-MET. However, AS1411-SL1-2 was capable of recruiting NCL, MDM2, and c-MET in concentration-dependent manners (**Figure S2X**). These results demonstrated that AS1411-SL1-2 induced the assembly of the MDM2-NCL-PROTAC-c-MET quaternary complex, employing NCL as a molecular bridge between MDM2 and c-MET. We examined whether the AS1411-SL1 chimeras-induced c-MET degradation was through the ubiquitin-proteasome pathway. The tandem ubiquitin-binding entities (TUBEs) pull-down and Co-IP assays showed that AS1411-SL1-2 or AS1411-SL1-3 increased ubiquitination of c-MET in MNNG/HOS cells **(Figure 2R and 2S)**. Further, the proteasome inhibitor MG132 heavily blocked c-MET degradation in MNNG/HOS cells treated with AS1411-SL1-2 or AS1411-SL1-3 (**Figure 2T**). We also determined whether the lysosomal pathway was involved in AS1411-SL1 chimeras-induced c-MET degradation. Bafilomycin A1 (BafA1), a lysosomal inhibitor, mildly reduced c-MET degradation in MNNG/HOS cells treated with AS1411-SL1-2 or AS1411-SL1-3 (**Figure S2Y**). We determined whether the c-MET degradation induced by AS1411-SL1-2 and AS1411-SL1-3 was dependent on the presence of NCL and MDM2. Silencing of NCL or MDM2 abolished the effect of AS1411-SL1-2 or AS1411-SL1-3 on c-MET degradation (**Figure 2U and 2V**).

### Antitumor activity of AS1411-SL1 chimeras *in vitro*

**Figure 3:**
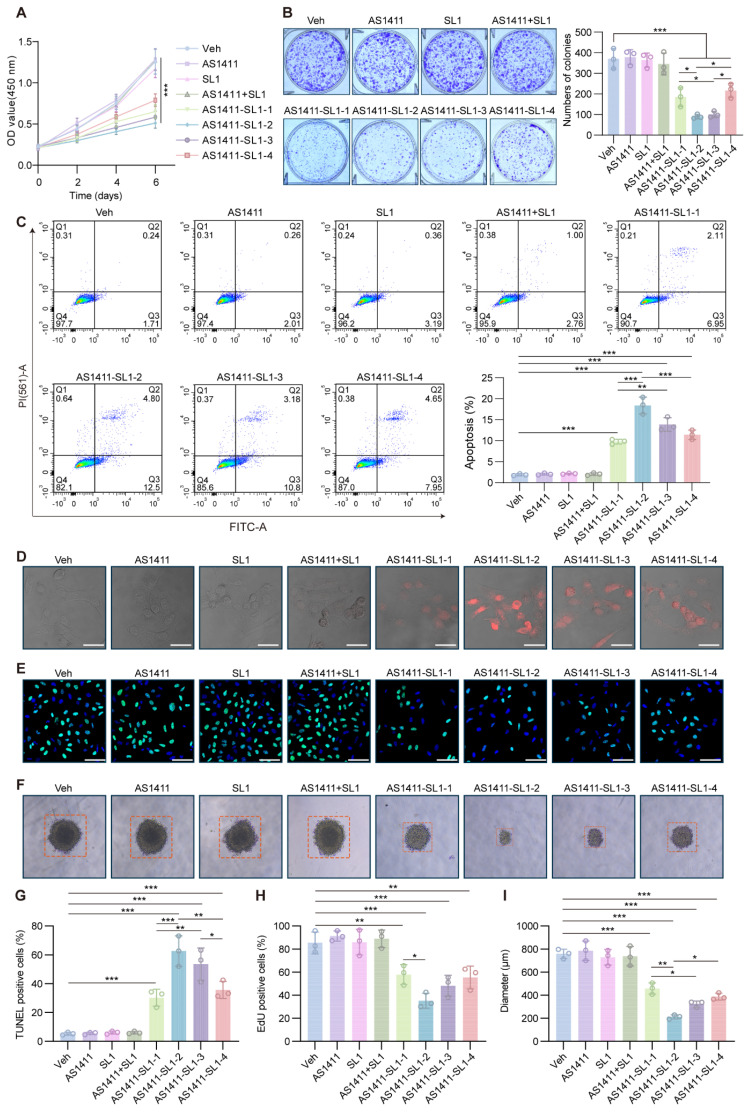
***In vitro* antitumor activity of AS1411-SL1 molecules.** (**A**) CCK-8 assay for the viability of MNNG/HOS cells after treatment with Veh, SL1, AS1411, AS1411-SL1-1, AS1411-SL1-2, AS1411-SL1-3, AS1411-SL1-4 or AS1411+SL1 every day at a concentration of 500 nM for 6 days. (**B**) Colony formation assay of MNNG/HOS cells after treatment with Veh, SL1, AS1411, AS1411-SL1-1, AS1411-SL1-2, AS1411-SL1-3, AS1411-SL1-4 or AS1411+SL1 every two days at a concentration of 500 nM for 8 days. Left, colony formation images; right, quantification of the colony formation. (**C**) Representative scatter plots (left) and quantification (right) of the apoptosis of MNNG/HOS cells after 5-day treatment with Veh, SL1, AS1411, AS1411-SL1-1, AS1411-SL1-2, AS1411-SL1-3, AS1411-SL1-4 or AS1411+SL1 every day at a concentration of 500 nM, as determined by flow cytometry. (**D**) TUNEL assay detecting apoptosis of MNNG/HOS cells treatment with Veh, SL1, AS1411, AS1411-SL1-1, AS1411-SL1-2, AS1411-SL1-3, AS1411-SL1-4 or AS1411+SL1 every day at a concentration of 500 nM for 3 days. Scale bar, 50 μm. (**E**) Edu incorporation assay showing proliferation of MNNG/HOS cells after treatment with Veh, SL1, AS1411, AS1411-SL1-1, AS1411-SL1-2, AS1411-SL1-3, AS1411-SL1-4 or AS1411+SL1 every day at a concentration of 500 nM for 3 days. Nuclei of MNNG/HOS cells were counterstained with DAPI. (**F**) Representative images of spheroids of MNNG/HOS cells after 5-day treatment with Veh, SL1, AS1411, AS1411-SL1-1, AS1411-SL1-2, AS1411-SL1-3, AS1411-SL1-4 or AS1411+SL1 every day at a concentration of 500 nM. (**G-I**) Quantification of the TUNEL-positive cells (**G**), Edu-positive cells (**H**), and diameters of spheroids of MNNG/HOS cells (**I**) under different treatments. Data were presented as mean ± SD. Each of the above experiments was repeated three times. *P*-values from one-way ANOVA (C, G, H, I) or two-way ANOVA (A): *P < 0.05, **P < 0.01, ***P < 0.001.

To investigate the antitumor potential of the AS1411-SL1 chimeras *in vitro*, we incubated MNNG/HOS cells with AS1411, SL1, AS1411-SL1-1, AS1411-SL1-2, AS1411-SL1-3, AS1411-SL1-4 or AS1411+SL1. CCK-8 assays and colony formation analyses revealed that all four chimeras (AS1411-SL1-1, AS1411-SL1-2, AS1411-SL1-3, and AS1411-SL1-4) significantly inhibited the proliferation of OS cells, whereas AS1411, SL1, and AS1411+SL1 had no inhibitory effect on OS cell growth (**Figure 3A and 3B**). Flow cytometry assays demonstrated that all four AS1411-SL1 chimeras increased the apoptotic rate of MNNG/HOS cells (**Fig. 3C**). Consistently, TUNEL and EdU staining suggested that all four chimeras promoted apoptosis and inhibited the proliferation of MNNG/HOS cells (**Fig. 3D and 3E**). In a 3D culture setting, we observed that all four chimeras reduced the size of MNNG/HOS spheroids (**Fig. 3F**). We performed quantitative analysis of the TUNEL and EdU staining and spheroid formation of MNNG/HOS cells. The quantitative results validated the antitumor activity of all four chimeras (**Fig. 3G-3I**). Among the four chimeras, AS1411-SL1-2 and AS1411-SL1-3 demonstrated superior antitumor ability *in vitro* (**Fig. 3A-3I**). We also incubated DU145 and HeLa cells with the AS1411-SL1 chimeras and demonstrated that the AS1411-SL1 chimeras decreased growth and increased apoptosis of DU145 and HeLa cells (**Fig. S3A-S3D**).

### Tumor-targeting property of the AS1411-SL1 chimeras *in vivo*

**Figure 4:**
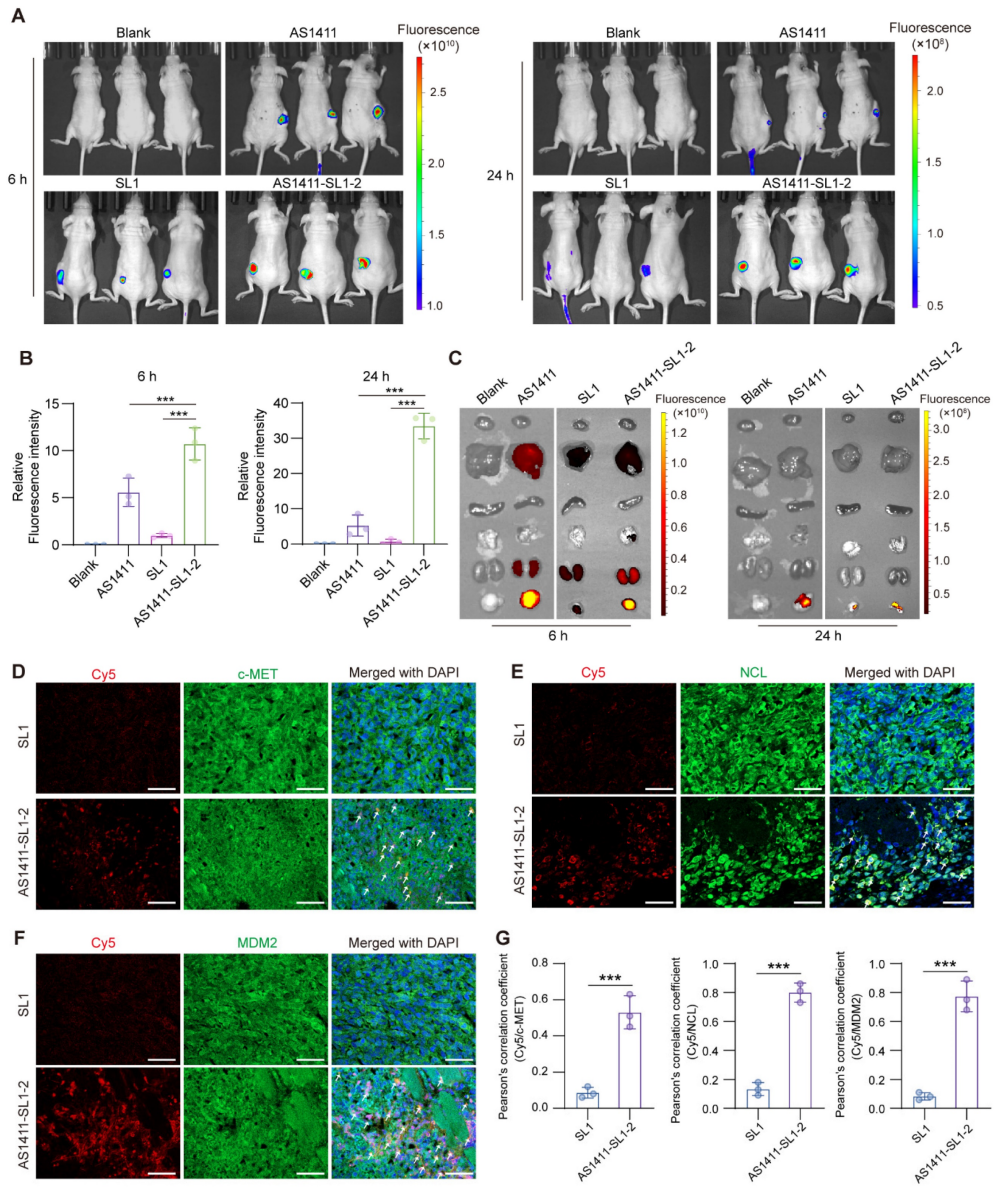
***In vivo* distribution of AS1411-SL1-2.** (**A**) Biophotonic images showing the whole-body distribution of AS1411, SL1, or AS1411-SL1-2 in nude mice bearing MNNG/HOS xenograft tumors. Each of the nude mice was intravenously injected with 5 nmol AS1411, SL1, or AS1411-SL1-2 for 6 or 24 h before the *in vivo* imaging analysis. AS1411 or SL1 was labeled with Cy5. Cy5-labeled SL1 was then conjugated to AS1411 to generate the Cy5-labeled AS1411-SL1-2. The untreated mice served as the blank control. (**B**) Quantitative analysis of the tumor distribution of Cy5-labeled AS1411, SL1, or AS1411-SL1-2 at 6 or 24 h. (**C**) *Ex vivo* fluorescence images of the xenograft tumors and major organs (heart, liver, spleen, lung, and kidney) from the mice after injection of Cy5-labeled AS1411, SL1, or AS1411-SL1-2 at 6 or 24 h. (**D-F**) Immunofluorescent staining showing *in vivo* co-localization of Cy5-labeled SL1 or AS1411-SL1-2 with c-MET (**D**), NCL (**E**), or MDM2 (**F**) in the xenograft tumors from the nude mice injected with SL1 or AS1411-SL1 for 6 h. Nuclei of MNNG/HOS cells were counterstained with DAPI. Arrows indicated the co-localization of SL1 or AS1411-SL1-2 with c-MET, NCL, or MDM2. SL1 or AS1411-SL1-2 was shown in red. c-MET, NCL, or MDM2 was shown in green. Cell nuclei were shown in blue. Scale bars, 100 μm. (**G**) Quantification of the co-localization of SL1 or AS1411-SL1-2 with MDM2, NCL, or c-MET. Data were represented as mean ± SD. *P*-values from two-tailed t-test: ***P < 0.001. n = 3 for each group.

To evaluate the tumor-targeting property of AS1411-SL1-2 *in vivo*, each of the MNNG/HOS-xenografted nude mice was intravenously injected with 5 nmol single-dose Cy5-labeled AS1411, SL1, or AS1411-SL1-2 for 6 and 24 h. Whole-body biophotonic imaging and quantitative analyses revealed that there was no fluorescence signal in the blank group of mice at 6 or 24 h (**Figure 4A and 4B**). In line with the *in vitro* tumor-targeting studies, AS1411 demonstrated more pronounced fluorescence signals and sustained accumulation at the tumor sites compared to SL1, indicating a greater surface expression of NCL than c-MET on OS cells. AS1411-SL1-2 showed enhanced tumor distribution than either AS1411 or SL1 alone, possibly due to the combined effects of AS1411 and SL1 (**Figure 4A and 4B**). Further, the *ex vivo* fluorescence imaging results showed that there was more tumor accumulation of AS1411-SL1-2 than AS1411 and SL1 (**Figure 4C**). Interestingly, fluorescence signals were minimal in the heart, spleen, and lungs in all treatment groups (**Figure 4C**). Next, we investigated whether there was co-localization of AS1411-SL1-2 with c-MET, NCL or MDM2 in OS cells *in vivo*. Confocal imaging revealed significant aggregation of fluorescent signals in OS cells of mice injected with AS1411-SL1-2, but little signals were seen in tumor sections of mice treated with SL1 (**Figure 4D-4G**). Notably, the fluorescence of AS1411-SL1-2 within OS cells exhibited obvious co-localization with c-MET, NCL, and MDM2 (**Figure 4D-4G**).

### Antitumor activity of AS1411-SL1 chimeras in a subcutaneous xenograft model

**Figure 5:**
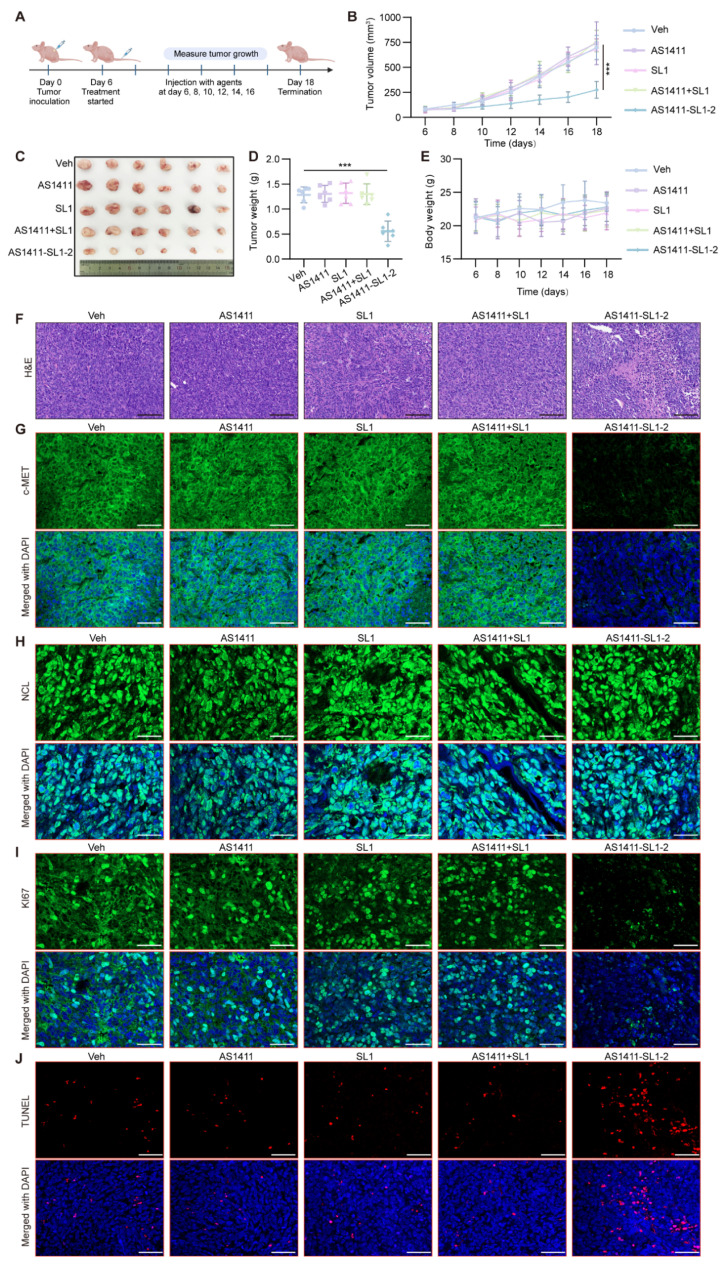
***In vivo* antitumor efficacy of AS1411-SL1-2 in a subcutaneous xenograft tumor model.** (**A**) Illustration of the experimental design for determining the antitumor efficacy of AS1411-SL1-2 in a subcutaneous xenograft tumor mouse model. Briefly, nude mice were subcutaneously injected with 2 × 10^6^ MNNG/HOS cells. After 6 days, the mice bearing xenograft tumors were intravenously administrated with Veh, SL1, AS1411, AS1411+SL1, or AS1411-SL1-2 for 12 days at a dose of 3 μmol/kg every two days. (**B**) The volume of the xenograft tumors from the nude mice in each treatment group. (**C**) Images of the xenografted tumors from the nude mice in each treatment group. (**D**) Weight of the xenograft tumors from the nude mice in each treatment group. (**E**) Changes in the body weight of the mice bearing xenograft tumors in each treatment group. (**F**) Hematoxylin and eosin (H&E) staining of the tumor sections from the nude mice in each treatment group. Scale bars = 100 μm. (**G-I**) Immunofluorescent staining of the tumor sections for detecting the levels of c-MET (**G**), NCL (**H**), or Ki-67 (**I**) in each treatment group. (**J**) TUNEL assay for detection of apoptotic cells in the tumor sections from the nude mice in each treatment group. Scale bars = 100 μm, Data were presented as mean ± SD. *P*-values from one-way ANOVA (D) or two-way ANOVA (B, E): ***P < 0.001. n = 6 for each group.

To examine the antitumor activity of AS1411-SL1-2 *in vivo*, each of the MNNG/HOS-xenografted mice was continuously treated with Veh, SL1, AS1411, AS1411-SL1-2, or AS1411+SL1 (a physical mixture of AS1411 and SL1) every two days via tail-vein injection at a dose of 3 μmol/kg (**Figure 5A**). After 12-day treatment, the average volume of tumor tissues from mice treated with AS1411-SL1-2 was smaller than that of the tumor tissues from other groups (**Figure 5B**). There was a marked reduction of tumor size in mice treated with AS1411-SL1-2 (**Figure 5C and 5D**). No significant change in weight was observed in all the treatment groups (**Figure 5E**). Hematoxylin and eosin (H&E) staining showed a higher incidence of apoptotic and necrotic cells in tumor sections from mice treated with AS1411-SL1-2 (**Figure 5F**). We further examined the level of c-MET and NCL in tumor tissues and observed that AS1411-SL1-2 effectively degraded c-MET while leaving NCL level unaffected (**Figure 5G and 5H**). Ki-67 staining and terminal deoxynucleotidyl transferase dUTP nick end labeling (TUNEL) assay revealed fewer Ki-67-positive cells and more TUNEL-positive cells in tumor tissues from the AS1411-SL1-2 group than the others (**Figure 5I and 5J**). We conducted serum biochemical assays to assess liver and kidney function parameters including ALT, AST, and BUN. The results showed no significant alterations in these parameters in mice treated with AS1411-SL1-2 when compared to other groups (**Figure S4A-S4C**). Histological analysis of major organs (heart, liver, spleen, lung, and kidney) indicated no significant damage to normal tissues caused by AS1411-SL1-2 (**Figure S4D and S4E**).

### Antitumor potency of AS1411-SL1 chimeras in an orthotopic xenograft model

**Figure 6:**
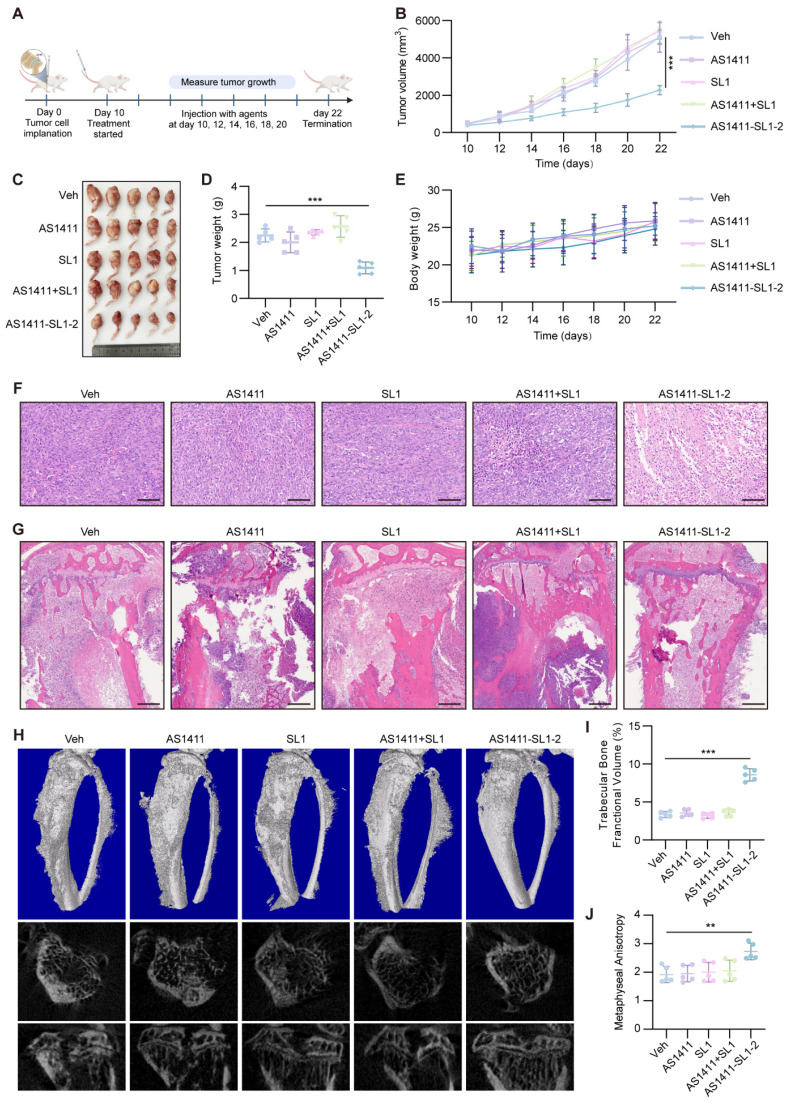
***In vivo* antitumor efficacy of AS1411-SL1-2 in an orthotopic xenograft model.** (**A**) Illustration of the experimental design for examining the antitumor efficacy of AS1411-SL1-2 in an orthotopic xenograft mouse model. Briefly, six-week-old SCID mice were inoculated with 3 × 10^5^ MNNG/HOS cells by intra-bone marrow administration. After 10 days, the SCID mice bearing orthotopic xenograft OS were intravenously administrated with Veh, SL1, AS1411, AS1411+SL1, or AS1411-SL1-2 for 12 days at a dose of 3 μmol/kg every two days. (**B**) The volume of the orthotopic xenograft OS from the SCID mice in each treatment group. (**C**) Images of the orthotopic xenograft OS from the SCID mice in each treatment group. (**D**) Weight of the orthotopic xenograft OS from the SCID mice in each treatment group. (**E**) The changes in body weight of the SCID mice bearing orthotopic xenograft OS in each treatment group. Scale bars, 100 μm. (**F**) H&E staining of the orthotopic xenograft OS sections from the SCID mice in each treatment group. Scale bars, 100 μm. (**G**) H&E-stained sections of bone and extraosseous soft tissues from the SCID mice bearing orthotopic xenograft OS in each treatment group. Scale bars, 500 μm. (**H**) Representative μCT images of the proximal tibia from the SCID mice bearing orthotopic xenograft OS in each treatment group. (**I**) Relative tibia metaphyseal bone fractional volume (BV/TV) of the SCID mice bearing orthotopic xenograft OS in each treatment group. (**J**) Anisotropic measurements from tibial metaphyses of the SCID mice bearing orthotopic xenograft OS in each treatment group. Data were presented as mean ± SD. *P*-values from one-way ANOVA (D, I, J) or two-way ANOVA (B, E): **P < 0.01, ***P < 0.001, n = 5 for each group.

We employed an orthotopic OS xenograft mice model to assess the antitumor efficacy and safety of AS1411-SL1-2. Each of the mice bearing orthotopic xenograft tumors was intravenously administrated with Veh, SL1, AS1411, AS1411-SL1-2, or AS1411+SL1 every two days at a dose of 3 μmol/kg (**Figure 6A**). After 12-day treatment, there was a significant reduction in the tibial xenograft tumors from mice treated with AS1411-SL1-2 when compared to other treatments (**Figure 6B-6D**). Body weight of mice bearing orthotopic xenograft tumors was not changed by AS1411-SL1-2 (**Figure 6E**). A higher incidence of apoptotic and necrotic cells and inhibited OS growth was observed in the mice treated with AS1411-SL1-2. (**Figure 6F and 6G**). Micro-computed tomography (μCT) analysis demonstrated attenuated bone destruction in mice treated with AS1411-SL1-2 (**Figure 6H-6J**). Serum biochemical assays confirmed no substantial changes in liver and kidney function parameters (ALT, AST, and BUN) in mice treated with AS1411-SL1-2 (**Figure S5A-S5C**). There was no significant damage to the major organs in mice treated with AS1411-SL1-2 (**Figure S5D and S5E**).

## Discussion

Most recently, we have conjugated AS1411 with ligands for a series of oncogenic POIs, such as STAT3, c-Myc, p53-R175H, and AR-V7 40. These AS1411-based PROTACs could selectively recognize and penetrate tumor cells, leading to ubiquitination and proteasomal degradation of the POIs in tumor cells 40. Obviously, all these POIs are intracellular proteins that have been considered hotspot targets of PTOTACs. It has been reported that PROTAC has rarely been used for the design of degraders of membrane POIs 45. In this study, we utilized AS1411 to create dual aptamers-functionalized PROTACs for degrading cell membrane c-MET, leading to targeted therapy of OS.

We conducted comprehensive studies to evaluate whether our AS1411-SL1 chimeras offered advantages to overcome drawbacks of traditional c-MET inhibitors or degraders, such as drug resistance and side effects. Our findings revealed that AS1411-SL1 chimeras specifically induced c-MET degradation in tumor cells without influencing c-MET level in normal cells, thereby demonstrating the tumor-targeting ability of the chimeras. This was consistent with the tumor-selective accumulation and good safety profile of AS1411-SL1 chimeras *in vivo*, which might be attributed to the absence of NCL on the surface of normal cells and no binding of AS1411 with normal cells. Among the four AS1411-SL1chimeras, AS1411-SL1-2 and AS1411-SL1-3 showed the most promising targeting capabilities for OS cells, aligning with their more potent antitumor effects compared to other chimeras *in vitro*.

Moreover, the AS1411-SL1 chimeras effectively degraded c-MET in tumor cells that were resistant to traditional inhibitors. Conceptually, our AS1411-SL1 chimeras fall under a category of bridged PROTAC technology 46, wherein AS1411 engages MDM2 by using NCL as a molecular bridge. Therefore, it is crucial to investigate whether NCL could be degraded by MDM2. Our data indicated that NCL is not a substrate for MDM2. In fact, many proteins that interact with MDM2 have been identified, and these are categorized into two groups: one group consists of proteins that are substrates for MDM2, just like p53, while the other group includes proteins that act upstream of MDM2 and regulate its E3 ligase activity, such as AKT, SKI, 14-3-3σ, and NCL as well 47-49. Theoretically, other upstream regulators of MDM2 from the latter category could serve as alternative candidates to NCL for the development of diverse MDM2-harnessing bridged PROTACs.

Prior to our work, several studies have integrated aptamer technology with PROTACs 50-52. However, these studies primarily employ aptamers as targeting agents to facilitate cell-specific delivery of PROTACs or incorporate aptamers into PROTACs as targeting warheads of POIs 50-52. In our study, we used AS1411 as both a tumor-targeting moiety and an E3 ligase recruiter, which differs from the previous studies. Additionally, bispecific aptamer chimeras that simultaneously engage the cell-surface lysosome-shuttling receptor (insulin growth factor receptor II, IGFIIR) and target membrane-bound POIs have been developed as lysosome-targeting chimeras (LYTACs) 53. These chimeras have demonstrated efficacy in redirecting cell surface proteins like c-MET and PTK-7 to lysosomes for degradation. However, IGFIIR is frequently mutated or deleted in some malignant tumors and acts as a tumor suppressor 54, raising concerns about the lack of tumor specificity of these aptamer-based LYTACs. In contrast, our study presented a superior alternative with the development of AS1411-based dual aptamer-functionalized PROTACs. The inclusion of tumor-targeting AS1411 enabled our PROTACs to selectively recognize and infiltrate tumor cells, offering a strategic advantage over aptamer-based LYTACs dependent on IGFIIR. In elucidating the mechanism by which AS1411-SL1 chimeras modulated c-MET expression, our findings demonstrated that the chimeras predominantly promoted the ubiquitination and proteasomal degradation of c-MET in both NCL- and MDM2-dependent manners. Nevertheless, inhibition of the lysosomal pathway also mildly reduced the degradation of c-MET induced by AS1411-SL1 chimeras, corroborating earlier reports that AS1411-conjugated therapeutics were partially trafficked through the endo-lysosomal pathway 55.

In addition, an earlier study introduced a BAAP strategy, designed to selectively modulate the function of cell surface receptors, such as phosphorylation-driven activation of receptors 56. The strategy employs bispecific aptamer probes as molecular connectors, simultaneously binding a target receptor protein and a paired protein. This dual binding brings the two proteins into close proximity on the living cell membrane, leading to functional inhibition of the receptor via significant steric hindrance 56. Most notably, this work exemplified the BAAP strategy via developing a bispecific aptamer probe by conjugating AS1411 with SL1 56, mirroring our design for constructing AS1411-SL1 chimeras. However, this study showed that the probe effectively decreased the level of p-c-MET without affecting the total c-MET level 56, which seems contradictory to our results. To classify this, we repeated the same experiments under the identical conditions reported in the original study, particularly employing the specified concentration (100 nM) of the AS1411-SL1 chimeras for the incubation of tumor cells 56. Our replicated experiments confirmed that the chimeras at a concentration of 100 nM indeed only affected the p-c-MET level without degrading total c-MET, aligning with the published study 56. Inspired by these results, we also examined the level of p-c-MET in tumor cells following treatment with the AS1411-SL1 chimeras at varied concentrations ranging from 100 to 1000 nM. We discovered that AS1411-SL1 chimeras at a concentration of 100 or 200 nM significantly decreased the p-c-MET level rather than altering the level of total c-MET in OS cells, which was consistent with the published study 56. However, the degradation of total c-MET was observable when tumor cells were incubated with AS1411-SL1 chimeras at concentrations exceeding 500 nM. Collectively, our work does not contradict the previous study 56. Instead, our work, together with the published study 56, indicates that low concentrations of AS1411-SL1 chimeras may interfere with the activation of c-MET (p-c-MET), whereas high concentrations of AS1411-SL1 chimeras are required to trigger the c-MET degradation. These fascinating findings call for further investigation to elucidate the underlying mechanisms. We boldly speculate that these findings may be linked to an earlier opinion that increased AS1411 could cause hyperstimulation of micropinocytosis in tumor cells and provoke enhanced cellular uptake 57, thus facilitating AS1411-SL1 chimeras-induced intracellular degradation of c-MET via a ubiquitin-proteasome pathway, rather than simply modulating p-c-MET level on the tumor cell surface.

Interestingly, our c-MET PROTACs were pure nucleic acid molecules, as the c-MET ligand SL1, the MDM2-recruiting AS1411, and the linkers between SL1 and AS1411 were DNA oligonucleotides. Regarding the linkers, we used double-stranded DNA sequences composed of A-T base pairs. Theoretically, a G-C base pair is more stable than an A-T base pair because it forms three hydrogen bonds, creating a stronger interaction between the bases, compared to the two hydrogen bonds of the A-T pair 58. However, AS1411 is a G-rich DNA oligonucleotide capable of folding into a highly polymorphic G-quadruplex structure, which confers increased resistance to degradation by serum nucleases and fluctuations in pH 59. Thus, we selected A-T base pairs as the linkers in our AS1411-SL1 PROTACs since a G-C base linker might disrupt the optimal formation of the G-quadruplex structure of AS1411. In our previous work, we confirmed the stability of the 6 A-T base pair linkers when we conjugated AS1411 with decoy oligonucleotides for transcriptional factors (TF) to create AS1411-based TF-PROTACs 40. Thus, we also chose the 6 or 10 A-T base pair linkers for constructing the AS1411-SL1 chimeras in this study. In addition, we were worried about whether the PROTACs consisting of AS1411 and SL1 could lead to the desired degradation of c-MET, given that both AS1411 and SL1, as well as the linkers, had larger molecular weights than conventional small molecular PROTACs. However, our results demonstrated that the AS1411-SL1 PROTACs were highly efficient degraders of c-MET and resulted in remarkable OS shrinkage and attenuation of bone destruction with no obvious toxicity. This could be attributed to the NCL-mediated rapid internalization of the AS1411-SL1 PROTACs in a tumor cell-specific manner.

Despite the inherent serum nuclease resistance of the AS1411 aptamer due to its G-quadruplex structure 59, we still observed the decreased stability of AS1411-SL1 chimeras following serum incubation. We speculated that this might be attributed to the serum instability of the SL1 aptamer component in the chimeras 60. The serum degradation of the AS1411-SL1 chimeras provides a possible explanation for the partially restored level of c-MET protein in OS cells upon treatment with the chimeras for 18 or 24 h. To enhance the effectiveness of the AS1411-SL1 chimeras in c-MET degradation, it is crucial to consider comprehensive chemical modifications in future studies. These modifications include the capping of the 3' end with inverted thymidine or biotin, incorporation of phosphorothioate or methylphosphonate groups into the phosphate backbone, introduction of 2'-sugar modifications such as fluoro, amino, or O-methyl groups, as well as the application of locked nucleic acid technology 61. Such modifications are anticipated to significantly improve the serum stability of the AS1411-SL1 chimeras. After those modifications, some follow-up experiments can be performed, such as investigations into the long-term stability and efficacy of the dual aptamer-functionalized PROTAC degraders and exploration of the potential off-target effects and safety profile of the degraders.

In summary, we have developed novel tumor-targeting PROTACs that selectively degrade membrane c-MET, which balances the therapeutic efficacy and potential toxicity. This study presents a significant advancement in targeted therapy by offering a potent and precise approach to combat OS using the innovative PROTAC technology. AS1411-based PROTACs are promising modulators to fill gaps for degrading membrane POIs, displaying advantages over traditional small molecular degraders.

## Materials and Methods

### Cell culture

The MNNG/HOS (TCHu167) cell line purchased from the National Collection of Authenticated Cell Cultures (China) was cultured in Eagle's Minimum Essential Medium (EMEM, ATCC, USA) supplemented with 10% fetal bovine serum (FBS). The DU-145 (HTB-81) and HeLa (CRM-CCL-2) cell lines obtained from the American Type Culture Collection (ATCC, USA) were maintained in Dulbecco's Modified Eagle's Medium (DMEM, Corning, USA) with a 10% FBS addition. The Hs746T cell line purchased from LYNJUNE (China) was maintained in Dulbecco's Modified Eagle's Medium (DMEM, Corning, USA) with a 10% FBS addition. The MC3T3-E1 cell line obtained from the American Type Culture Collection (ATCC, USA) and MLO-Y4 cell line purchased from UBIGENE (China) were maintained in Minimum Essential Medium α (α-MEM, Corning, USA). All cells were incubated at 37 °C in a humidified atmosphere containing 5% CO_2_.

### Construction of PROTACs

DNA oligonucleotides, including CRO, AS1411, and SL1, were synthesized from Sangon Biotech Co., Ltd (Shanghai, China). PROTAC degraders for c-MET were constructed as follows: the 3' terminus of AS1411 was concatenated with the sense strand of the double-stranded DNA linker (6 or 10 T), and the 3' terminus or 5' terminus of SL1 was concatenated with the anti-sense strands of the double-strand DNA linker (6 or 10 A). The concatenated sequences were heated at 95°C for 5 min, followed by 37°C for 30 min, and then mixed at a molar ratio of 1:1 at 4°C overnight. AS1411: 5'-GGTGGTGGTGGTTGTGGTGGTGGTGG-3'. CRO: 5'-CCTCCTCCTCCTTCTCCTCCTCCTCC-3'. SL1: 5'-ATCAGGCTGGATGGTAGCTCGGTCGGGGTGGGTGGGTTGGCAAGTCTGAT-3'. Double-stranded DNA linker consisting of 6 A-T base pairs: 5'-TTTTTT-3'/5'-AAAAAA-3'. Double-stranded DNA linker consisting of 10 A-T base pairs: 5'-TTTTTTTTTT-3'/5'-AAAAAAAAAA-3'.

### Stability of PROTAC degraders

PROTAC degraders were incubated with 10% FBS or cell lysates at 37°C for 1, 3, 6, 9, 12, and 24 h. Post-incubation, samples were mixed with DNA loading dye (Sangon, China) and run on non-denaturing PAGE at 100 V for 2 h. The gel was then stained with 4S Gelblue (Sangon, China) for 20 min at room temperature and visualized under UV light.

### RNA interference

Cells were grown in 6-well plates until they reached 60-80% confluency. siRNAs were diluted in an Opti-MEM medium (Gibco, USA). Lipofectamine RNAiMAX reagent (Invitrogen, USA) was also diluted in Opti-MEM and rested for 5 min at room temperature. Both solutions were then combined and incubated for an additional 10 min at room temperature. This mixture was added to the cells for transfection.

### Western blot

Western blot was performed as previously described 62. Briefly, total proteins were separated by SDS-PAGE gel and transferred onto a polyvinylidene fluoride (PVDF) membrane (Millipore, USA) using a Trans-Blot Turbo^TM^ (Bio-Rad, USA). Subsequently, the PVDF membrane was blocked with a solution of 5% non-fat dry milk in TBS-T for 1 h at room temperature. After blocking, the membrane was incubated overnight at 4°C with primary antibodies (anti-c-MET, 1:1000, CST; anti-NCL, 1:2000, CST; anti-Tubulin, 1:5000, Abclonal; anti-p-c-MET, 1:1000, anti-MDM2, 1:1000, anti-ERK, 1:2000, anti-p-ERK, 1:1000, anti-AKT, 1:2000, anti-p-AKT, 1:1000, anti-MST1R, 1:1000, Proteintech). The membrane was washed three times with TBS-T, followed by incubation with the appropriate HRP-conjugated secondary antibodies for 1 h at room temperature. The blots were developed using an enhanced chemiluminescence ECL kit (ABclonal, China) and imaged with a chemiluminescence imaging system (Tanon, Multi5200, CN).

### Sample preparation and LC-MS/MS Analysis

Cell extracts were prepared using the EasyPep Mini MS Sample Prep Kit (Thermo Scientific, USA) as per the manufacturer's instructions. Briefly, 100 μg of protein sample was treated with reduction and alkylation solutions, followed by digestion with Trypsin/Lys-C Protease Mix solution. The digested peptides were then purified and reconstituted with 0.1% formic acid in water for LC-MS/MS analysis. The analysis was performed using an Orbitrap Fusion Lumos Tribrid Mass Spectrometer (Thermo Scientific, USA) coupled with an Easy-nLC 1000 (Thermo Scientific, USA) ultrahigh-pressure liquid chromatography pump. The peptides were separated into a trap column and an analytical column packed with C18 resins. Full MS scans were acquired over an m/z range of 395-1205, and data analysis was performed using Spectronaut (version 14.9, Biognosys, CH).

### Spheroid growth assay

MNNG/HOS cells were seeded in ultra-low attachment, round-bottom, 96-well plates (Corning, USA) to promote spheroid formation. Half of the medium in each well was replaced daily to maintain optimal growth conditions. After different treatments, the spheroids were imaged using a microscope (Nikon, ECLIPSE Ts2, JP).

### Cell viability assay

Cell viability was assessed using a CCK-8 assay (MCE, USA). Cells were seeded in 96-well plates and incubated overnight for adherence. After different treatments, 10 μL of CCK-8 reagent and 90 μL of medium were added to each well. Following a 1.5-hour incubation, absorbance at 450 nm was measured using a PerkinElmer EnSpire® spectrometer (USA).

### Colony formation assay

Cells were plated in 6-well plates and incubated overnight to ensure adherence. After different treatments, cells were fixed in 10% formalin (Solarbio, China) and stained with crystal violet (Beyotime, China) for 20 min at room temperature. Digital imaging of the resultant colonies was performed, and colony enumeration was conducted using ImageJ software version 10.3.

### Immunofluorescence staining

Tumor sections of 5-μm thickness underwent deparaffinization using xylene. Subsequently, the sections were rehydrated and subjected to antigen retrieval. After antigen retrieval and blocking with QuickBlock™ Blocking Buffer (Beyotime, China), sections were incubated overnight at 4°C with primary antibodies (anti-Ki67, 1:100, CST; anti-c-MET, 1:100, CST; anti-NCL,1:100, anti-MDM2, 1:100, Proteintech) and secondary antibodies. Finally, the tissue sections were mounted with a mounting medium containing DAPI (Abcam, UK). Images were taken with a confocal fluorescence microscope (Zeiss, LSM980, DE).

### Co-IP assay

Co-IP assay was performed as previously described 63. Briefly, cells after different treatments were lysed using IP lysis buffer (Thermo Fisher Scientific, USA) supplemented with a proteinase inhibitor cocktail (NCE, China). After centrifugation at 13,000 × g at 4 °C, the supernatant was incubated with the corresponding primary antibody overnight at 4 °C and then coupled to Protein A/G Magnetic Beads (Thermo Fisher Scientific, USA) at room temperature for 1 h. To reduce nonspecific binding, the beads were extensively washed five times using a DynaMag™-2 Magnet (Thermo Fisher Scientific, USA). The immunoprecipitated complexes were then resuspended in a loading buffer, heated at 95 °C for 5 min, and subsequently analyzed by SDS-PAGE and western blotting.

### Pull-down assay

Cells were incubated with biotin-labeled CRO, AS1411 or the AS1411-SL1 chimeras, followed by lysis using IP lysis buffer (Thermo Fisher Scientific, USA) supplemented with a proteinase inhibitor cocktail (NCE, China). The lysate supernatants were then incubated with streptavidin-coated magnetic beads (Thermo Fisher Scientific, USA) at 4 °C overnight. After five washes, the beads were resuspended in a loading buffer and boiled for 5 min. Finally, the proteins from the pull-down assay and input samples were analyzed using SDS-PAGE and western blotting.

### Binding assay of AS1411

MNNG/HOS cells were suspended in an FBS-free medium and incubated with Cy5-labeled CRO, AS1411, or the AS1411-SL1 chimeras at room temperature. After washing, a flow cytometer (BD, FACSCanto SORP, USA) was used to analyze the binding ability of CRO, AS1411, or the AS1411-SL1 chimeras with the cells. Data analysis was conducted using the FlowJo software package, with gating specifically set to single cells.

### Apoptosis assay

Cells were plated in 6-well plates and incubated overnight to ensure adherence. After different treatments, they were detached using a non-EDTA cell dissociation buffer (Beyotime, China). Apoptotic cells were stained using an Annexin V-FITC Apoptosis Detection Kit (Beyotime, China). Flow cytometric analysis was conducted using a BD FACSCanto SORP (USA), with data analysis carried out via the FlowJo software.

### Biophotonic imaging analysis

After administration with Cy5-labeled AS1411, SL1, or AS1411-SL1, the mice were anesthetized, and whole-body fluorescent imaging was conducted using the IVIS® Spectrum system (PerkinElmer, USA). Post-imaging, the mice were euthanized, and their tumors and major organs (heart, lung, liver, spleen, and kidney) were isolated for biphotonic imaging. All images were captured with a 1-second exposure time, and the fluorescence distribution was visualized as pseudo-color images both *in vivo* and *ex vivo*.

### Animals

Male BALB/c nude mice and SCID mice aged 6-8 weeks were purchased from the GemPharmatech Co., Ltd (Guangdong, China). These mice were accommodated within the Laboratory Animal House at the Southern University of Science and Technology, which maintained a controlled temperature environment and a 12-hour light/dark cycle. Food and water were available* ad libitum*. All *in vivo* experimental protocols received approval from the Institutional Animal Care and Use Committee (IACUC) of the Southern University of Science and Technology.

### Subcutaneous xenograft model

A subcutaneous MNNG/HOS tumor xenograft model was established as described above 64. Briefly, BALB/c nude mice were subcutaneously engrafted on the right dorsal flank with 2 × 10^6^ MNNG/HOS cells. After 6 days, these mice were monitored for tumor volume and body weight. Meanwhile, mice were injected intravenously with different treatments every two days. On day 18, the mice were sacrificed, and tumors were excised and weighed.

### Orthotopic xenograft model

Each SCID mice received intra-bone marrow injections of 3 × 10^5^ MNNG/HOS cells. Before the inoculation, mice were anesthetized with 2% isoflurane (RWD, China). To facilitate the injection, the left hind limb was positioned at a 90° angle, and a 27 G needle was used to penetrate the tibial plateau, delivering 10 μL of cell suspension. After 10 days, these mice were monitored for tumor volume and body weight. Meanwhile, mice were injected intravenously with different treatments every two days. On day 22, these mice were sacrificed. Primary tumors and the adjacent affected bone were excised and weighed* en bloc*. The femur was severed proximal to the tumor using scissors, while the tibia was cut at its distal end.

### μCT analysis

Briefly, fixed non-demineralized bone was subjected to μCT analysis using a Bruker μCT (SkyScan 1276, Belgium) with a 60KV X-ray source and 100uA current at a resolution of 10 μm. Scanned images were reconstructed by NRecon software, and a three-dimensional model was constructed by CTvox software with the same thresholds for each sample.

### H&E staining

Tissue sections underwent deparaffinization in xylene and were progressively rehydrated through a graded series of ethanol to water. Following this, they were stained using standard H&E protocols. After the staining process, the sections were covered with coverslips. The final specimens were then imaged using an Aperio GT 450 scanner (Leica, DE).

### Serum biochemical assays

Following treatment, blood samples were obtained and key serum biochemical parameters, including alanine aminotransferase (ALT), aspartate aminotransferase (AST), and blood urea nitrogen (BUN), were quantitatively assessed using an MS-480 automatic Biochemistry Analyzer (Medicalsystem Biotechnology, CN).

### TUNEL assay

Tumor sections underwent deparaffinization in xylene and were progressively rehydrated through a graded ethanol series to water. Next, they were incubated with a mixture of terminal deoxynucleotidyl transferase enzyme and Cy3-labeled dUTP from the One Step TUNEL Apoptosis Assay Kit (Beyotime) at 37°C for 1 h. After incubation, the sections were mounted using a DAPI-containing medium (Abcam, UK). Fluorescent images of the prepared sections were captured using a Zeiss LSM980 confocal microscope (DE).

### ELISA

To quantify the concentration of eluted proteins from the biotin pull-down assay, a quantitative sandwich ELISA was conducted using kits specific for MDM2 (Abnova), NCL (LS Bio), and c-MET (Thermo Fisher Scientific), following the manufacturer's instructions. In brief, assay buffer, samples, and an antibody cocktail were added to a pre-coated 96-well microplate and incubated at room temperature. After washing, the detection solution and stop solution were applied. Absorbance was measured at 450 nm or 595nm using a spectrophotometer (PerkinElmer, EnSpire®, USA).

### SPR assays

All experiments were conducted using a Biacore 8K instrument (Biacore AB, GE Healthcare) and CM5 sensor chips (catalog number 29149603, Revvity). All solutions were filtered using 0.22 µm Millipore filters. Target proteins were immobilized on the sensor chip surface via amine groups following a standard procedure. Briefly, the chip matrix was activated by injecting a 1:1 (vol/vol) mixture of 100 mM NHS and 400 mM EDC for 10 minutes at a flow rate of 10 µL/min. Target proteins, diluted in 10 mM acetate buffer (catalog number BR100349, Revvity), were then injected at a concentration of 20 µg/mL and coupled for 5 minutes at a flow rate of 5 µL/min. After immobilization, the remaining reactive groups on the chip surface were blocked by injecting 1 M ethanolamine for 10 minutes at a flow rate of 10 µL/min. Compounds were serially diluted in HBS buffer (10 mM HEPES, pH 7.4, 0.137 M NaCl, 3.4 mM EDTA, and 0.05% P20). The diluted compounds were injected for 180 seconds, followed by a dissociation phase of 180 seconds at a flow rate of 30 µL/min. At the end of each cycle, the sensor chip surface was regenerated by injecting 100 mM glycine/HCl (pH 2.0) for 30 seconds at a flow rate of 30 µL/min. All data were analyzed using Biacore Insight Evaluation Software (Version 2.0.15.12933).

### Statistical analysis

All numerical data are expressed as the mean ± SD. A two-sided unpaired t-test was employed to compare the two groups. Comparisons among multiple groups were analyzed using one-way ANOVA followed by Dunnett's test. To assess differences in cell viability, tumor volume, and body weight between groups, a two-way ANOVA was performed, followed by Tukey's multiple comparisons test. P < 0.05 was considered statistically significant. All statistical analyses were performed with GraphPad Prism 9 software. We chose the representative images based on the average/median level of the data for each group. For the *in vivo* experiments, the sample size was pre-determined by a power calculation. The mice were grouped randomly and blindly by researchers. The mice in poor body condition before the experiments were excluded.

## Supplementary Material

Supplementary figures and table.
