# Supplementary material for: Development of dual aptamers-functionalized c-MET PROTAC degraders for targeted therapy of osteosarcoma
Source: Theranostics. 2025 Jan 1;15(1):103–21. doi: 10.7150/thno.99588 (PMC11667235; doi:10.7150/thno.99588)
Supplement: Supplementary file 1 — Supplementary figures and table. [file thnov15p0103s1.pdf]

Supplementary Information

Development of dual aptamers-functionalized c-MET PROTAC degraders for targeted therapy of osteosarcoma

Xuekun Fu[1,#], Jie Huang[1,2,#], Xinxin Chen[1], Duoli Xie[1,2], Hongzhen Chen[1], Zhijian Liang[1], Zhuqian Wang[1,2], Yiying Liang[3], Aiping Lu[2,4,5*], Chao Liang[1,2,6,*]

[1]Department of Systems Biology, School of Life Sciences, Southern University of Science and Technology, Shenzhen 518055, China.

[2]Institute of Integrated Bioinfomedicine and Translational Science (IBTS), School of Chinese Medicine, Hong Kong Baptist University, Hong Kong SAR 999077, China.

[3]Shenzhen LingGene Biotech Co., Ltd., Shenzhen 518055, China.

[4]Guangdong-Hong Kong-Macau Joint Lab on Chinese Medicine and Immune Disease Research, Guangzhou 510006, China.

[5]Shanghai University of Traditional Chinese Medicine, Shanghai 200032, China.

[6]State Key Laboratory of Proteomics, National Center for Protein Sciences (Beijing), Beijing Institute of Lifeomics, Beijing 100850, China.

[#]These authors contributed equally.

**Correspondence: aipinglu@hkbu.edu.hk (A.L.), liangc@sustech.edu.cn (C.L.)*

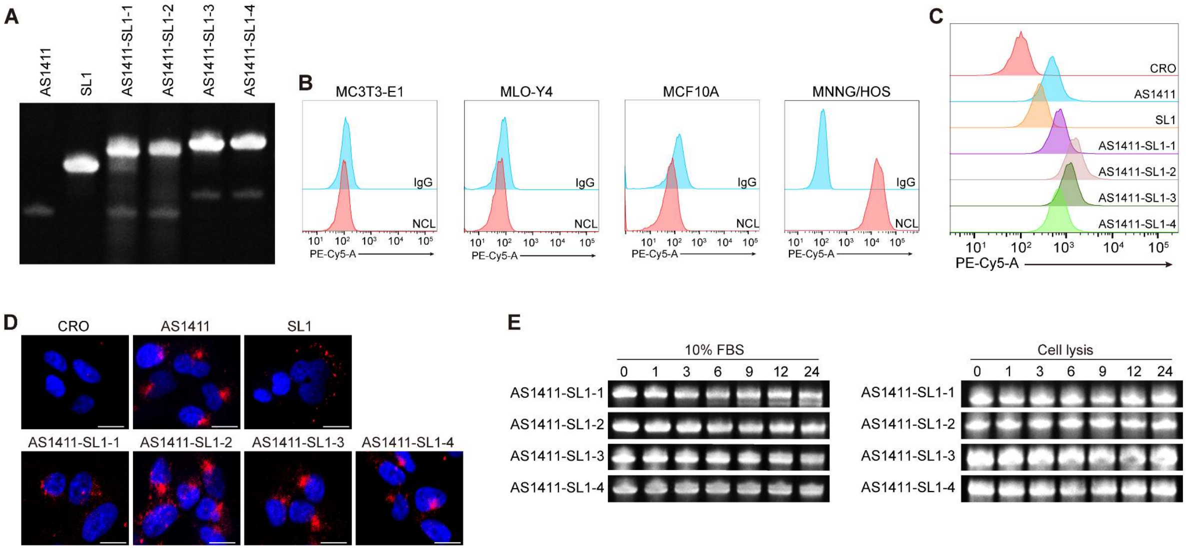

Figure S1. The characterization and binding assay of the AS1411-SL1 chimeras *in vitro*.

(**A**) Nondenaturing polyacrylamide gel electrophoresis (PAGE) of AS1411, SL1, AS1411-SL1-1, AS1411-SL1-2, AS1411-SL1-3 and AS1411-SL1-4. (**B**) Expression of NCL on the surface of an OS cell line MNNG/HOS, an osteoblast precursor cell line MC3T3-E1, an osteocyte-like cell line MLO-Y4, and a non-malignant epithelial cell line MCF 10A, as determined by flow cytometric analysis using an anti-NCL antibody or an IgG isotype control. (**C**) Flow cytometry assay depicting the binding of 500 nM Cy5-labeled CRO, AS1411, SL1, AS1411-SL1-1, AS1411-SL1-2, AS1411-SL1-3, and AS1411-SL1-4 with MNNG/HOS cells for 2 h. (**D**) Fluorescence images showing the internalization of 500 nM Cy5-labeled CRO, AS1411, SL1, AS1411-SL1-1, AS1411-SL1-2, AS1411-SL1-3, and AS1411-SL1-4 into MNNG/HOS cells after incubation for 6 h. Scale bar, 10 μm. (**E**) Stability of AS1411-SL1-1, AS1411-SL1-2, AS1411-SL1-3, and AS1411-SL1-4 in 10% FBS (left) or in MNNG/HOS cell lysates (right) at the indicated time points (0, 1, 3, 6, 9, 12, and 24 h), as determined by non-denaturing PAGE. Each of the above experiments was repeated three times.

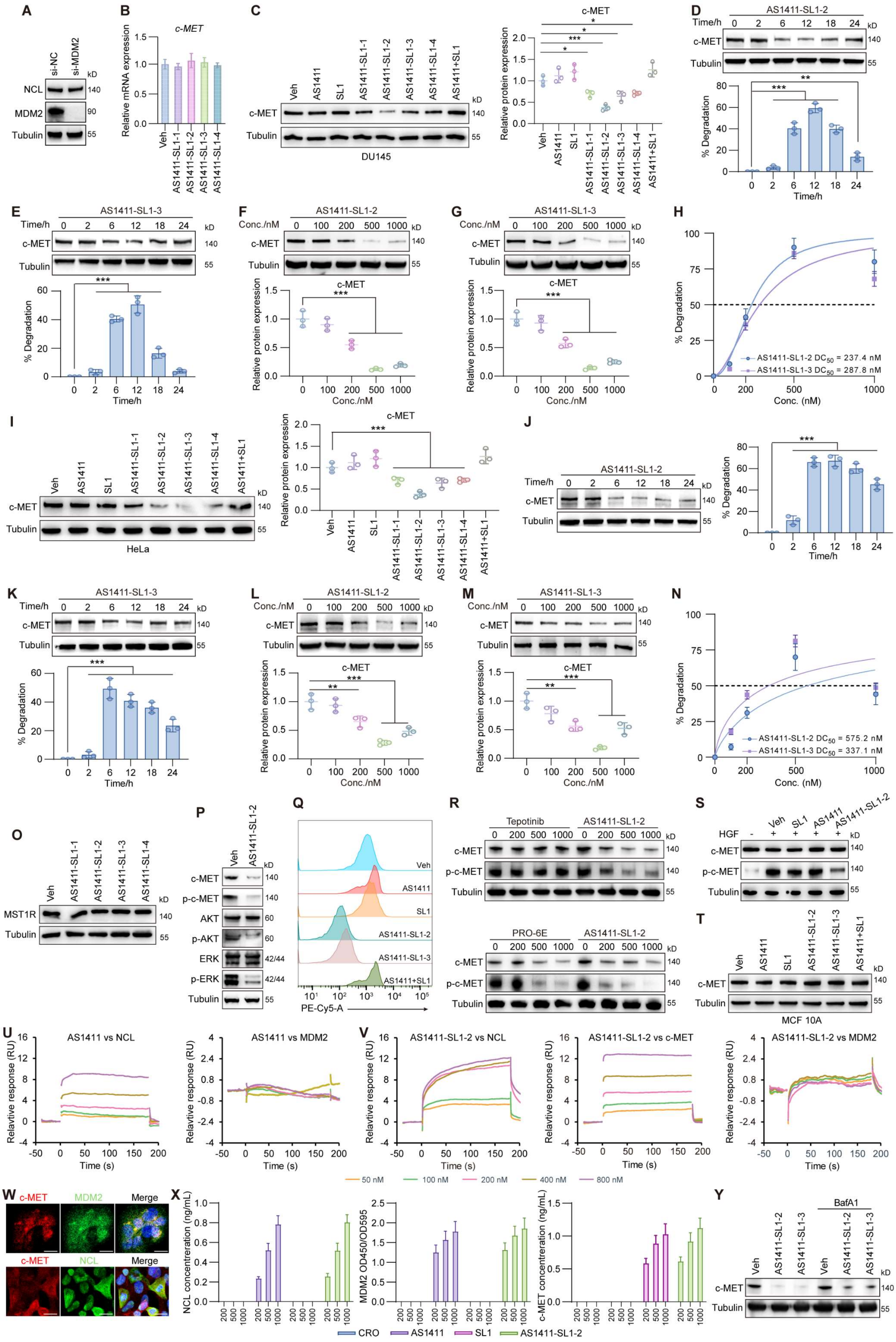
A
si-NC
si-MDM2
NCL
MDM2
Tubulin
B
c-MET
Relative mRNA expression
Veh
AS1411-SL1-1
AS1411-SL1-2
AS1411-SL1-3
AS1411-SL1-4
C
AS1411
SL1
AS1411+SL1
DU145
Relative protein expression
D
AS1411-SL1-2
Time/h
% Degradation
E
AS1411-SL1-3
F
Conc./nM
G
H
AS1411-SL1-2 DC50 = 237.4 nM
AS1411-SL1-3 DC50 = 287.8 nM
Conc. (nM)
I
HeLa
J
K
L
M
N
AS1411-SL1-2 DC50 = 575.2 nM
AS1411-SL1-3 DC50 = 337.1 nM
O
MST1R
P
p-c-MET
AKT
p-AKT
ERK
p-ERK
Q
PE-Cy5-A
R
Tepotinib
PRO-6E
S
HGF
T
MCF 10A
U
AS1411 vs NCL
AS1411 vs MDM2
Relative response (RU)
Time (s)
V
AS1411-SL1-2 vs NCL
AS1411-SL1-2 vs c-MET
AS1411-SL1-2 vs MDM2
50 nM
100 nM
200 nM
400 nM
800 nM
W
Merge
X
NCL concentreation (ng/mL)
MDM2 OD450/OD595
c-MET concentreation (ng/mL)
CRO
Y
BafA1

Figure S2. *In vitro* degradation of c-MET by AS1411-SL1 molecules.

(**A**) Level of NCL protein in siMDM2- or siNC-transfected MNNG/HOS cells. (**B**) Level of *c-MET* mRNA in MNNG/HOS cells after treatment with Veh, AS1411-SL1-1, AS1411-SL1-2, AS1411-SL1-3, or AS1411-SL1-4 at a concentration of 500 nM for 6 h. (**C**) Level of c-MET in a human prostate cancer cell line DU145 after treatment with Veh, AS1411, SL1, AS1411-SL1-1, AS1411-SL1-2, AS1411-SL1-3, AS1411-SL1-4, or AS1411+SL1 at a concentration of 500 nM for 6 h. Left, western blotting images; right, quantification of the c-MET protein. (**D** and **E**) Level of c-MET in DU145 cells after treatment with 500 nM AS1411-SL1-2 (**D**) or AS1411-SL1-3 (**E**) at the indicated time points (0, 2, 6, 12, 18, and 24 h). Top, western blotting images; bottom, quantification of the c-MET degradation. (**F** and **G**) Level of c-MET in DU145 cells after treatment with AS1411-SL1-2 (**F**) or AS1411-SL1-3 (**G**) at the indicated concentrations (0, 100, 200, 500, and 1000 nM) for 6 h. Top, western blotting images; left or bottom, quantification of the c-MET protein. (**H**) The DC_{50} values for AS1411-SL1-2 and AS1411-SL1-3 in DU145 cells. (**I**) Level of c-MET protein in a human cervical cancer cell line HeLa after treatment with Veh, AS1411, SL1, AS1411-SL1-1, AS1411-SL1-2, AS1411-SL1-3, AS1411-SL1-4 or AS1411+SL1 at a concentration of 500 nM for 6 h. Left, western blotting images; right, quantification of the c-MET protein. (**J** and **K**) Level of c-MET in HeLa cells after treatment with 500 nM AS1411-SL1-2 (**H**) or AS1411-SL1-3 (**I**) at the indicated time points (0, 2, 6, 12, 18, and 24 h). Top, western blotting images; bottom, quantification of the c-MET degradation. (**L** and **M**) Level of c-MET in HeLa cells after treatment with AS1411- SL1 -2 (**J**) or AS1411-SL1 -3 (**K**) at the indicated concentrations (0, 100, 200, 500, and 1000 nM) for 6 h. Top, western blotting images; bottom, quantification of the c-MET protein. (**N**) The DC_{50} values for AS1411-SL1-2 and AS1411-SL1-3 in HeLa cells. (**O**) Level of MST1R in MNNG/HOS cells after treatment with Veh, AS1411-SL1-1, AS1411-SL1-2, AS1411-SL1-3, and AS1411-SL1-4 at a concentration of 500 nM for 6 h. (**P**) Levels of c-MET, p-c-MET, AKT, p-AKT, ERK, and p-ERK in MNNG/HOS cells after treatment with 500 nM AS1411-2 for 6 h. (**Q**) Flow cytometry assay detecting the surface expression of c-MET on MNNG/HOS cells after the indicated treatments at a concentration of 500 nM for 12 h. (**R**) Levels of c-MET and p-c-MET in Hs746T^{D1228N} cells after treatment with tepotinib or AS1411-SL1-2 at the indicated concentrations (0, 200, 500, and 1000 nM) for 6 h (upper), or in MNNG/HOS cells after treatment with PRO-6E or AS1411-SL1-2 at the indicated concentrations (0, 200, 500, and 1000 nM) for 24 h (bottom). (**S**) Levels of c-MET and p-c-MET in MNNG/HOS cells after treatment with 100 nM SL1, AS1411, and AS1411-SL1-2 for 20 min followed by HGF stimulation for 30 min. (**T**) Level of c-MET in MCF 10A cells after treatment with Veh, AS1411, SL1, AS1411-SL1-2, AS1411-SL1-3, or AS1411+SL1 at a concentration of 500 nM for 6 h. **(U)** Surface plasmon resonance (SPR) assays for determining interaction between AS1411 (50, 100, 200, 400, 800 nM) and NCL or MDM2. **(V)** SPR assays for determining interaction between AS1411-SL1-2 (50, 100, 200, 400, 800 nM) and NCL, c-MET or MDM2. **(W)** Fluorescence images showing the co-localization of c-MET with NCL and MDM2 in MNNG/HOS cells after treatment with 500 nM AS1411-SL1-2. (**X**) Pull-down assays in combination with ELISA for quantification of NCL, MDM2, and c-MET captured by biotin-labeled AS1411, SL1, and AS1411-SL1-2 at the indicated concentrations (200, 500, and 1000 nM). (**Y**) Level of c-MET in MNNG/HOS cells after treatment with Veh or 500 nM AS1411-SL1-2 or AS1411-SL1-3 for 6 h, in the presence or absence of 200 nM lysosomal inhibitor Bafilomycin A1 (BafA1). Each of the above experiments was repeated three times. Data were presented as mean ± SD. *P*-values from one-way ANOVA: $^{**}P < 0.05$, $^{**}P < 0.01$, $^{***}P < 0.001$.

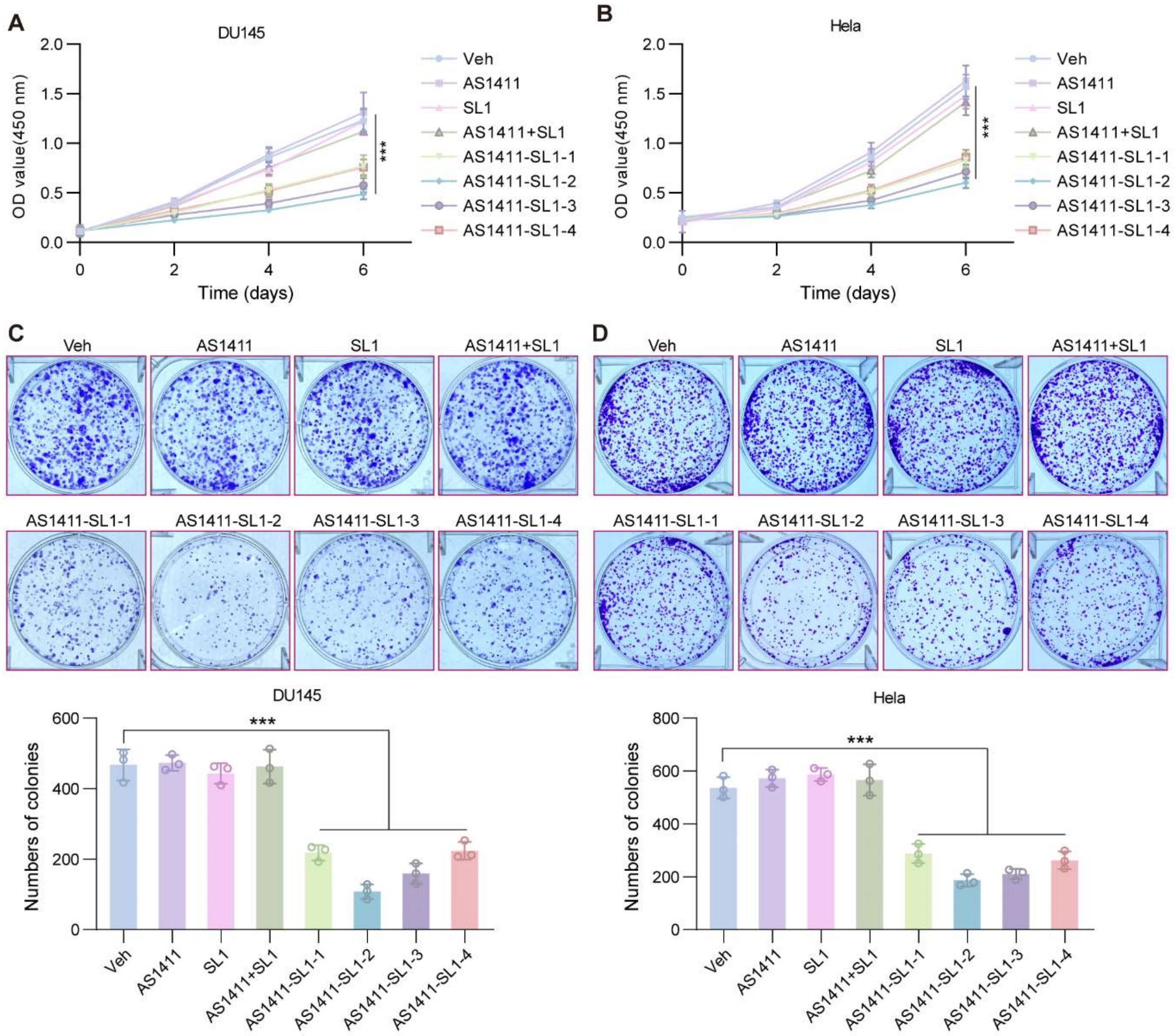

Figure S3. ***In vitro*** **antitumor activity of the AS1411-SL1 chimeras**

(**A** and **B**) CCK-8 assay for the viability of DU145 cells (**A**) or HeLa cells (**B**) after 6-day treatment with Veh, AS1411, SL1, AS1411-SL1-2, AS1411-SL1-3, or AS1411+SL1 every day at a concentration of 500 nM. (**C** and **D**) Colony formation assay of DU145 cells (**C**) or HeLa cells (**D**) after treatment with Veh, AS1411, SL1, AS1411-SL1-2, AS1411-SL1-3, or AS1411+SL1 every two days at a concentration of 500 nM for 8 days. Top, colony formation images; bottom, quantification of the colony formation images. Each of the above experiments was repeated three times. Data were presented as mean ± SD. *P*-values from one-way ANOVA: ****P* < 0.001.

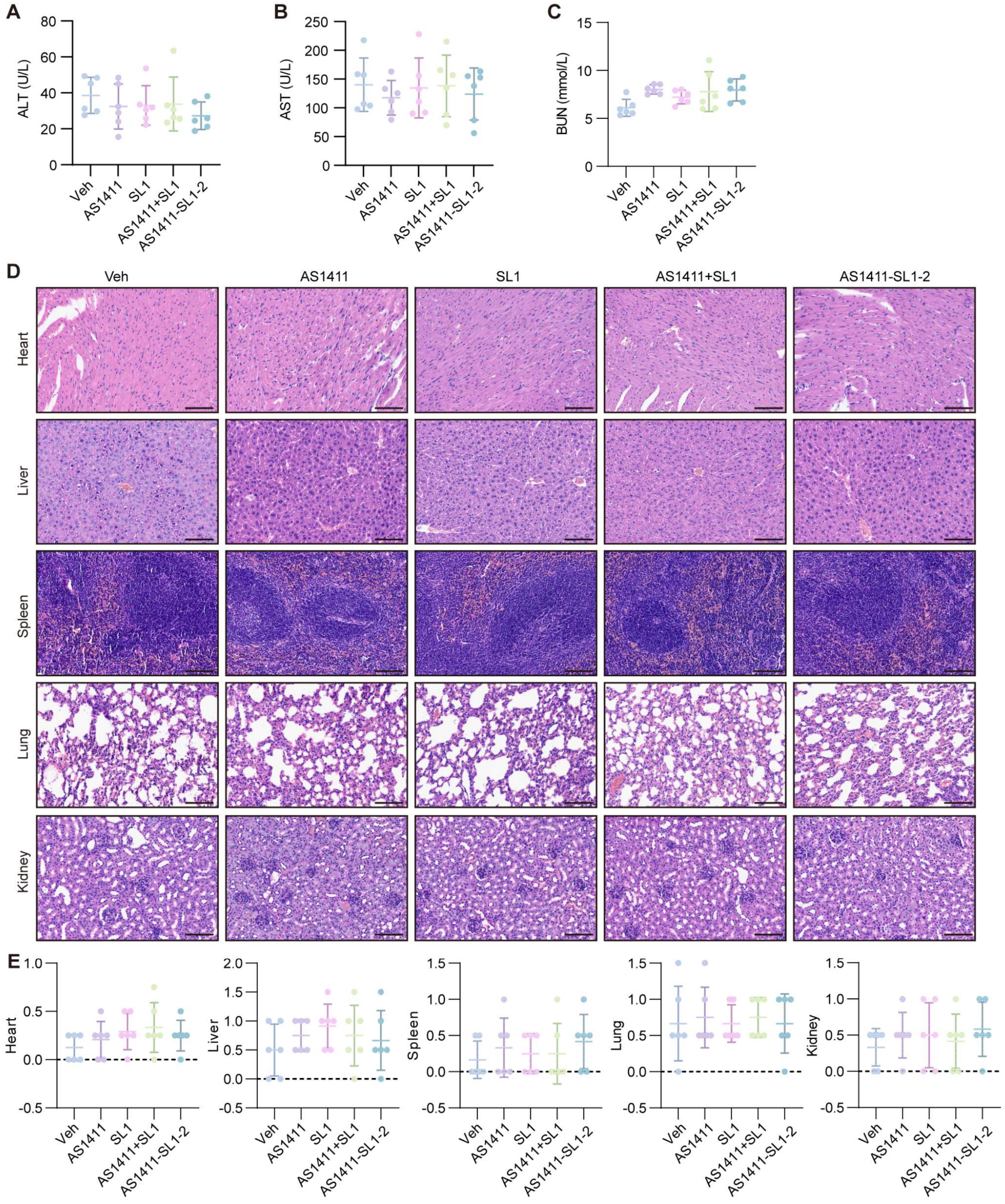

Figure S4. *In vivo* safety of AS1411-SL1-2 in a subcutaneous xenograft tumor model.
(**A-C**) Levels of serum alanine transaminase (ALT) (**A**), aspartate transaminase (AST) (**B**), or blood urea nitrogen (BUN) (**C**) in the nude mice bearing subcutaneous xenograft tumors after intravenous administration with Veh, SL1, AS1411, AS1411+SL1, or AS1411-SL1-2 for 12 days at a dose of 3 μmol/kg every two days, as determined by the automated hematology analyzer. (**D**) Histological assessments of major organs (heart, liver, spleen, lung, and kidney) from the nude mice in each treatment group by H&E staining. (**E**) Semi-quantitative histological analysis of the H&E-stained sections of the major organs from the nude mice in each treatment group. Data were presented as mean ± SD. n = 6 for each group.

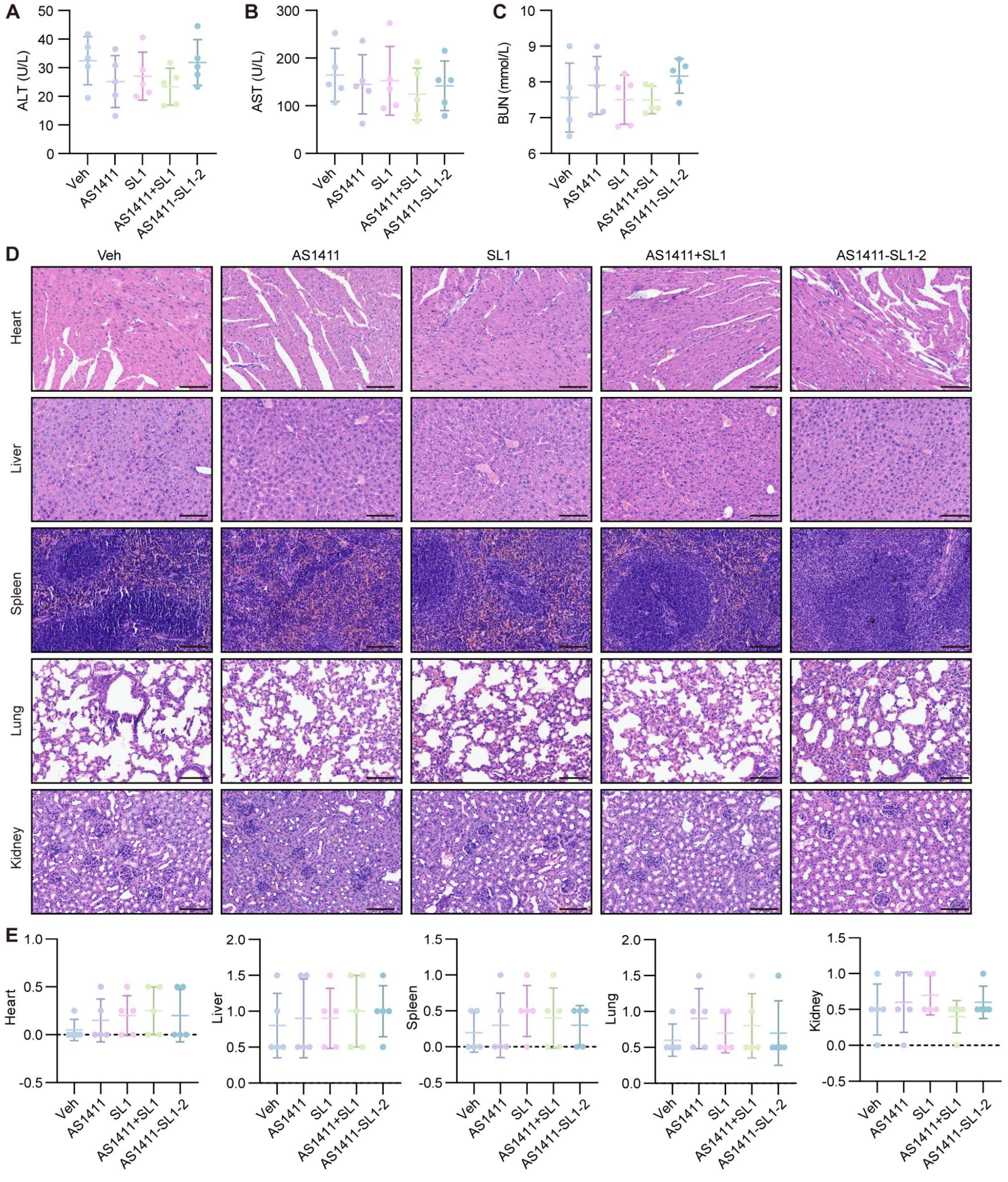

Figure S5. *In vivo* safety of AS1411-SL1-2 in an orthotopic xenograft model.

(**A-C**) The Levels of serum ALT (**A**), AST (**B**), or BUN (**C**) in the SCID mice bearing orthotopic xenograft tumors after intravenous administration with Veh, SL1, AS1411, AS1411+SL1, or AS1411-SL1-2 for 12 days at a dose of 3 μmol/kg every two days, as determined by the automated hematology analyzer. (D) Histological assessments of the major organs (heart, liver, spleen, lung, and kidney) from the SCID mice in each treatment group by H&E staining. (**E**) Semi-quantitative histological analysis of the H&E-stained sections of the major organs from the SCID mice in each treatment group. Data were presented as mean ± SD. n = 5 for each group.

Table S1 Other differentially expressed proteins in proteomic analysis

Protein Name	Protein Functions	Relationships to c-MET
AVL9	Involved in vesicle-mediated transport and cell migration	c-MET signaling can influence cell migration and vesicle transport [1]
BANP	DNA binding, chromatin organization	c-MET signaling can affect chromatin structure [2]
MCF2	Ras-GTPase activating protein, involved in cell proliferation	c-MET can activate Rho GTPase pathways [3]
PMPCA	Mitochondrial protein processing	c-MET signaling can influence cellular metabolism and mitochondrial function [4]
STXBP2	Involved in vesicle fusion	c-MET may influence vesicle transport indirectly [1]
SLC25A3	Mitochondrial phosphate carrier	c-MET signaling can influence mitochondrial function and metabolism [5]
NISCH	Cytoskeleton regulation, cell migration	c-MET signaling can influence cell morphology and movement [6]
RAP2A	a small GTPase involved in signaling pathways	c-MET can activate small GTPase pathways [7]
FRYL	Involved in cell differentiation	c-MET may influence cell differentiation indirectly [8]
CTHRC1	Cell migration	c-MET signaling can influence extracellular matrix remodeling and cell migration [9]
BAZ2A	Chromatin remodeling	c-MET signaling can affect chromatin structure and gene expression [10]
APC	a tumor suppressor involved in Wnt signaling and cell adhesion	c-MET signaling can interact with Wnt signaling and influence cell adhesion [11]
RAB3D	Vesicle transport	c-MET signaling can influence vesicle trafficking and secretion processes [1]
ERBB2	Receptor tyrosine kinase, tumor growth	ERBB2 and c-MET may co-regulate signaling pathways [12]
DUSP1	Dual specificity phosphatase, MAPK pathway regulation	c-MET signaling can regulate MAPK pathways [13]
DDX60L	Helicase, antiviral immune response	cMET signaling can affect immune responses [14]
DHX58	RIG-I-like receptor, antiviral immune response	c-MET signaling can modulate immune pathways [14]
ATG2B	Autophagy regulation	c-MET may indirectly influence autophagy [15]
RASA1	Ras-GTPase activating protein, regulates Ras signaling	c-MET can activate RAS pathways [16]

LAMP2	Lysosomal function, autophagy	c-MET may indirectly influence autophagy [17]
EI24	Apoptosis-inducing factor	c-MET regulates apoptosis [17]

References

1. Kermorgant S, Zicha D, Parker PJ. PKC controls HGF-dependent c-Met traffic, signalling and cell migration. The EMBO journal. 2004; 23: 3721-34-34.
2. Trusolino L, Bertotti A, Comoglio PM. MET signalling: principles and functions in development, organ regeneration and cancer. Nature reviews Molecular cell biology. 2010; 11: 834-48.
3. Liu J, Li S, Chen S, Chen S, Geng Q, Xu D. c-Met-dependent phosphorylation of RhoA plays a key role in gastric cancer tumorigenesis. The Journal of pathology. 2019; 249: 126-36.
4. Rodríguez-Hernández MA, de la Cruz-Ojeda P, López-Grueso MJ, Navarro-Villarán E, Requejo-Aguilar R, Castejón-Vega B, et al. Integrated molecular signaling involving mitochondrial dysfunction and alteration of cell metabolism induced by tyrosine kinase inhibitors in cancer. Redox Biology. 2020; 36: 101510.
5. Duplaquet L, Leroy C, Vinchent A, Paget S, Lefebvre J, Vanden Abeele F, et al. Control of cell death/survival balance by the MET dependence receptor. eLife. 2020; 9: e50041.
6. Birchmeier C, Birchmeier W, Gherardi E, Vande Woude GF. Met, metastasis, motility and more. Nature Reviews Molecular Cell Biology. 2003; 4: 915-25.
7. Rodrigues GA, Park M, Schlessinger J. Activation of the JNK pathway is essential for transformation by the Met oncogene. The EMBO journal. 1997; 16: 2634-45.
8. Boccaccio C, Andò M, Tamagnone L, Bardelli A, Michieli P, Battistini C, et al. Induction of epithelial tubules by growth factor HGF depends on the STAT pathway. Nature. 1998; 391: 285-8.
9. Viticchiè G, Muller PAJ. c-Met and Other Cell Surface Molecules: Interaction, Activation and Functional Consequences. Biomedicines. 2015; 3: 46-70.
10. Lai AZ, Abella JV, Park M. Crosstalk in Met receptor oncogenesis. Trends Cell Biol. 2009; 19: 542-51.
11. Gherardi E, Birchmeier W, Birchmeier C, Vande Woude G. Targeting MET in cancer: rationale and progress. Nature reviews Cancer. 2012; 12: 89-103.
12. Paulson AK, Linklater ES, Berghuis BD, App CA, Oostendorp LD, Paulson JE, et al. MET and ERBB2 are coexpressed in ERBB2+ breast cancer and contribute to innate resistance. Mol Cancer Res. 2013; 11: 1112-21.
13. Ponzetto C, Bardelli A, Zhen Z, Maina F, dalla Zonca P, Giordano S, et al. A multifunctional docking site mediates signaling and transformation by the hepatocyte growth factor/scatter factor receptor family. Cell. 1994; 77: 261-71.
14. Barbosa-Matos C, Borges-Pereira C, Libório-Ramos S, Fernandes R, Oliveira M, Mendes-Frias A, et al. Deregulated immune cell recruitment orchestrated by c-MET impairs pulmonary inflammation and fibrosis. Respiratory Research. 2024; 25: 257.
15. Nabeshima K, Inoue T, Shimao Y, Okada Y, Itoh Y, Seiki M, et al. Front-cell-specific expression of membrane-type 1 matrix metalloproteinase and gelatinase A during cohort migration of colon carcinoma cells induced by hepatocyte growth factor/scatter factor. Cancer research. 2000; 60: 3364-9.
16. Fixman ED, Fournier TM, Kamikura DM, Naujokas MA, Park M. Pathways downstream of Shc and Grb2 are required for cell transformation by the tpr-Met oncoprotein. The Journal of biological chemistry. 1996; 271: 13116-22.
17. Liu Y, Liu JH, Chai K, Tashiro S, Onodera S, Ikejima T. Inhibition of c-Met promoted apoptosis, autophagy and loss of the mitochondrial transmembrane potential in oridonin-induced A549 lung cancer cells. J Pharm Pharmacol. 2013; 65: 1622-42.
